# Broad-spectrum antibiotic prophylaxis in tumor and infected orthopedic surgery—the prospective-randomized, microbiologist-blinded, stratified, superiority trials: BAPTIST Trials

**DOI:** 10.1186/s13063-023-07605-5

**Published:** 2024-01-19

**Authors:** Ilker Uçkay, Hagen Bomberg, Markus Risch, Daniel Müller, Michael Betz, Mazda Farshad

**Affiliations:** 1 Department of Orthopedic Surgery, Balgrist University Hospital, University of Zurich, Forchstrasse 340, 8008 Zurich, Switzerland; 2 Unit for Clinical and Applied Research, Balgrist University Hospital, University of Zurich, Forchstrasse 340, 8008 Zurich, Switzerland; 3 Infection Control, Balgrist University Hospital, University of Zurich, Forchstrasse 340, 8008 Zurich, Switzerland; 4 Department of Anesthesiology, Intensive Care and Pain Medicine, Balgrist University Hospital, University of Zurich, Forchstrasse 340, 8008 Zurich, Switzerland; 5 Balgrist University Hospital, University of Zurich, Forchstrasse 340, 8008 Zurich, Switzerland

**Keywords:** Orthopedic surgery, Broad-spectrum antibiotic prophylaxis, Surgical site infections, Postoperative healthcare-associated infections, Randomized-controlled trial

## Abstract

**Background:**

The perioperative antibiotic prophylaxis with 1st or 2nd generation cephalosporins is evidence-based in orthopedic surgery. There are, however, situations with a high risk of prophylaxis-resistant surgical site infections (SSI).

**Methods:**

We perform a superiority randomized controlled trial with a 10% margin and a power of 90% in favor of the broad-spectrum prophylaxis. We will randomize orthopedic interventions with a high risk for SSI due to selection of resistant pathogens (open fractures, surgery under therapeutic antibiotics, orthopedic tumor surgery, spine surgery with American Society of Anesthesiologists (ASA) score ≥ 3 points) in a prospective-alternating scheme (1:1, standard prophylaxis with cefuroxime versus a broad-spectrum prophylaxis of a combined single-shot of vancomycin 1 g and gentamicin 5 mg/kg parenterally). The primary outcome is “remission” at 6 weeks for most orthopedic surgeries or at 1 year for surgeries with implant. Secondary outcomes are the risk for prophylaxis-resistant SSI pathogens, revision surgery for any reason, change of antibiotic therapy during the treatment of infection, adverse events, and the postoperative healthcare-associated infections other than SSI within 6 weeks (e.g., urine infections or pneumonia). With event-free surgeries to 95% in the broad-spectrum versus 85% in the standard prophylaxis arm, we need 2 × 207 orthopedic surgeries.

**Discussion:**

In selected patients with a high risk for infections due to selection of prophylaxis-resistant SSI, a broad-spectrum combination with vancomycin and gentamycin might prevent SSIs (and other postoperative infections) better than the prophylaxis with cefuroxime.

**Trial registration:**

ClinicalTrial.gov NCT05502380. Registered on 12 August 2022. Protocol version: 2 (3 June 2022)

**Supplementary Information:**

The online version contains supplementary material available at 10.1186/s13063-023-07605-5.

## Introduction

### Background and rationale

We investigate a possible superiority of a broad-spectrum antibiotic prophylaxis for selected orthopedic patients with an elevated risk of surgical site infections (SSI) due to multidrug-resistant pathogens. The perioperative antibiotic prophylaxis is a cornerstone in the prevention of deep SSI [1]. While the surgical debate regarding its duration (single versus triple-dose), double-dosing in obese patients [2], the pathogens to be targeted, or concerning the timing (before or after the intraoperative sampling) continues [2–9], there is less discussion for the choice of the antibiotic agents. In clean surgery such as the orthopedic field, most experts recommend the use of 2nd generation cephalosporins [1] or vancomycin in cases of allergy to β-lactam agents and/or skin colonization by methicillin-resistant *Staphylococcus aureus* [1, 6].

However, up to the half of all pathogens of the deep orthopedic SSIs are not covered by this cephalosporins [2–9] and reveal, for example, SSIs due to methicillin-resistant skin commensals [6] or Gram-negative, non-fermenting rods [5]. This high proportion of prophylaxis-resistant SSIs occurs in selected patient populations with orthopedic tumors [8, 9], open fractures [10], patient operated for wound dehiscence [11], ulcerated diabetic foot infections [3, 12], or in orthopedic patients that are operated under concomitant therapeutic antibiotic regimens [3, 13] for any reasons. In these populations, the therapeutic antibiotics are often much broader in the spectrum than the standard cephalosporin prophylaxes and tend to select more microorganisms in case of the occurrence of SSIs. Indeed, at least 10% of all new intraoperative tissue samples in orthopedic surgery, sampled under therapeutic antibiotics and open wounds, yield new pathogens that were previously unknown to the clinicians [3].

We call this a “selection” under antibiotic therapy or prophylaxis. The current antibiotics only kill the previously detected pathogens but left over newly introduced contaminants, especially if new microorganisms might penetrate into an unclosed wound. This selection leads to new SSIs occurring at the same orthopedic site [3]. However, and unfortunately, it is unpredictable to guess the future pathogens. These might be Gram-positive skin commensals or (multi)-resistant Gram-negative rods [3].

We ignore how to prevent selection in the aforementioned situations. Clinicians try with various approaches, for example, many patients under therapy of co-amoxiclav for aspiration pneumonia receive cefuroxime prophylaxis when operated, only because of existent and standardized protocols that advocate cefuroxime [1]. This makes no sense. Practically, no microorganism is covered more by cefuroxime than by co-amoxiclav. Other colleagues try to circumvent selection by routinely replacing cephalosporins by vancomycin in patients under therapeutic antibiotics or by vancomycin prophylaxis for patients with long hospitalization times prior to orthopedic surgery. However, this broadening of the prophylaxis only towards Gram-positive skin commensals regularly failed to show benefit in retrospective and prospective studies [4], because a substantial proportion of SSIs were Gram-negative [3–5]. From a microbiological perspective, only a maximal Gram-positive coverage, alongside with a large Gram-negative coverage, would cover most selections in these patient populations.

The BAPTIST Trial only concerns the perioperative antibiotic prophylaxis in selected orthopedic surgeries with a high risk of selection of prophylaxis-resistant SSI pathogens or of other healthcare-associated infections in the immediate postsurgical period: these are tumor surgery, surgery for dehiscent wounds, any orthopedic surgery under concomitant therapeutic antibiotics, open fractures, surgeries with skin colonization with multidrug-resistant bacteria, plus, as a control, orthopedic spine surgery in multimorbid patients. This spine surgery patients are closest to the study population in terms of demographic characteristics. We alternately randomize the standard prophylaxis (cefuroxime, clindamycin, or the current antibiotic treatment) to the broad-spectrum single-shot of vancomycin 1 g and single-shot of gentamicin 5 mg/kg intravenously. The rest of the medical and surgical interventions, the eventual use of negative-pressure vacuum therapy [14], the eventual use of topical anti-infective agents [15–17], and all other infection control measures remain unchanged [1, 18].

## Methods

### Study setting

The Balgrist University Hospital in Zurich is a tertiary, referral center for orthopedic surgery (including for orthopedic infections) and is affiliated to the University of Zurich. It presents a multi-disciplinary team composed of orthopedic surgeons, internists, specialized nurses and physiotherapists, musculoskeletal expert radiologists, and infectious diseases physicians who are specialized in orthopedic infections. Moreover, this team is accompanied by the Unit for Clinical and Applied Research with experience in investigative designs (www.balgrist.ch). The BAPTIST Trial starts at the Balgrist but is expandable to other (international) centers.

### Study objectives

We investigate if a perioperative broad-spectrum antibiotic prophylaxis would better prevent the occurrence of deep (prophylaxis-resistant) SSI, and/or the other bacterial nosocomial infections after the index surgery, when compared to standard prophylaxis in selected patient population at high risk for (prophylaxis-resistant) infections. We want to circumvent the potential selection of multi-resistant pathogens during their index surgery.

### Definitions and study outcomes

Tables 1 and 2 present key definitions and the outcomes of the trial. Briefly, an “orthopedic infection” requires the microbiological evidence of bacteria in at least two deep intraoperative tissue samples together with radiological (osteitis, collections, inflammation) and/or clinical evidence of infection (pus, discharge, sinus tracts, rubor, calor, pain). The presence of a histological proof is facultative. We define implants as any deep foreign material, except for allografts, transient wires, or fixator pins. “Remission” is the absence of clinical and/or radiological and/or laboratory signs of an eventual infection after a minimal follow-up time of 6 weeks for soft-tissue surgery or 1 year for implant-related surgery.

**Table 1:** Key study definitions

**Standard prophylaxis:**
1st–3rd generation cephalosporins, co-amoxiclav, clindamycin intravenously
**Broad-spectrum antibiotic prophylaxis:**
Vancomycin 1 g together with gentamicin 5 mg/kg intravenously
**Broad-spectrum antibiotic treatment:**
4–5th generation cephalosporins, quinolones, carbapenems, vancomycin, daptomycin, linezolid, aminoglycosides, colistin, metronidazole
**Remission**
No clinical, anamnestic, radiology, or laboratory signs for infection at the test-of-cure visit

**Table 2:** Outcome parameters and assessments of both randomized trials

**Primary outcome**
- Remission (and inversely deep surgical site infection) at 6 weeks for surgeries without implant and 1 year for surgeries with implant
**Secondary outcomes:**
- Risk of (antibiotic-resistant) pathogens in the deep surgical site of the study patients
- Revision surgery for non-infections reasons within 6 weeks
- Change of antibiotic therapy for infection, based on intraoperative findings
- Spectrum and adverse events of therapeutic antibiotic use (if any)
- Incidence and antibiotic resistance of non-SSI infections within 6 weeks (e.g., urine)
- Body colonization with multi-resistant bacteria at 4–-6 weeks (if any samples)

Assessment of outcomes: prospective assessment by study team during hospitalization. Retrospective assessment by study nurses and surgeons during the surgical controls after hospitalization. These controls are standard after 6 weeks and 1 year

### Interventions and study conduct

**Fig. 1:**
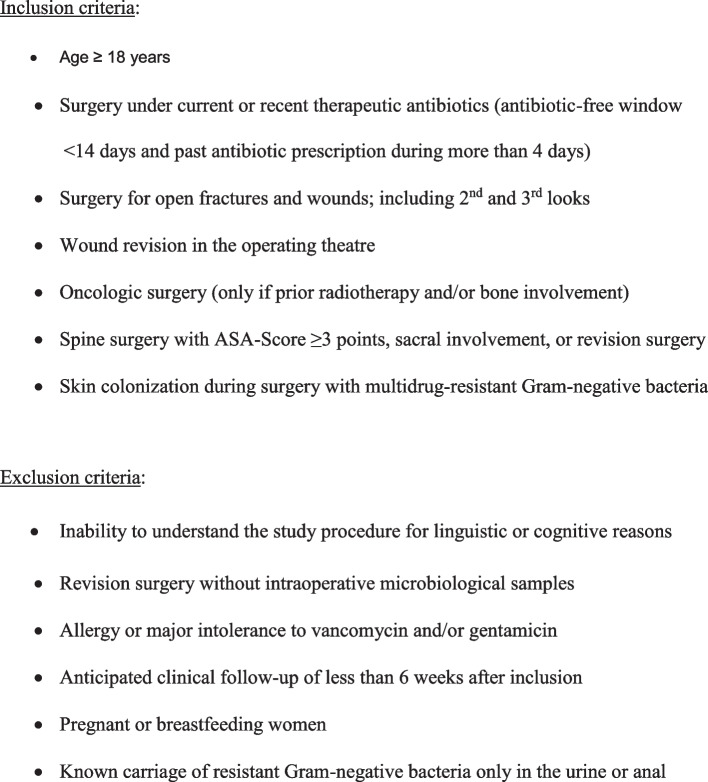
Study criteria

**Fig. 2:**
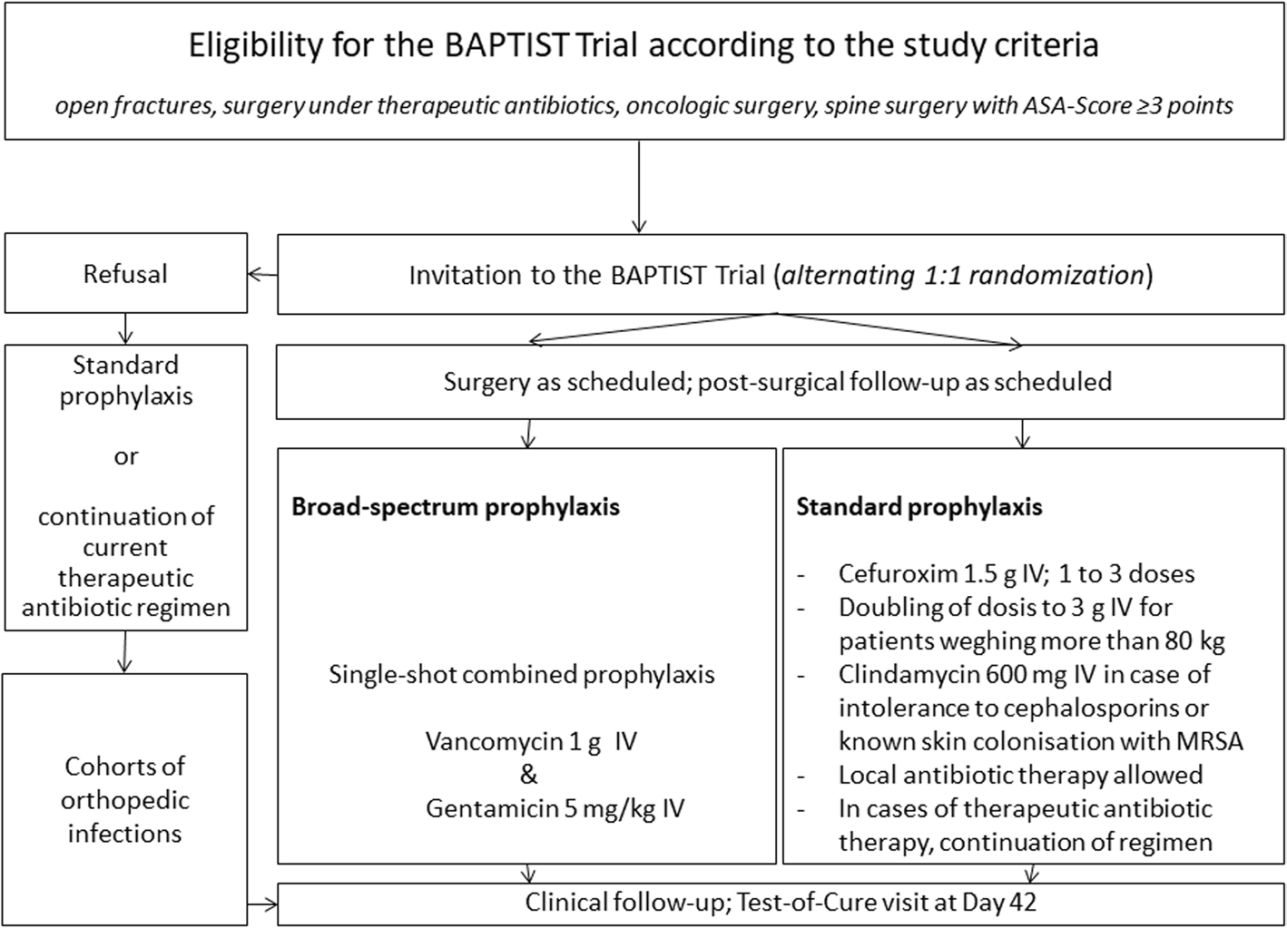
Main study flowchart

The study team screens all entries for potential study candidates on a daily basis, including the weekends and the night shifts. We randomize the high-risk surgeries in an alternating scheme (1:1) according to the scheduled position in our eight operating theatres. The anesthetists (or the nurses at the ward) administer the antibiotic prophylaxis immediately before surgery or after the intraoperative microbiological samples during surgery. After the prophylactic regimen, the clinicians are free to continue with any therapeutic antibiotic regimen, if there is suspicion of infection. Such a subsequent systemic or local (intraosseous) antimicrobial therapy per se is not an objective of the BAPTIST Trial. Likewise, we also allow other molecules to be (auto)administered by the patients, such as herbal medicine, homeopathic drugs, or skin antiseptic agents before surgery, e.g., in the frame of a presurgical decolonization. Figure 1 presents the inclusion and exclusion criteria and Fig. 2 the study flowchart. There will be no blinding of persons except for the microbiologist, and there will be no placebos. The Pharmacy of the Balgrist supplies all antibiotics.

During the study, we assess the following variables:

- *Patient’s characteristics*: age, sex, immune-suppression (diabetes, renal dialysis, cirrhosis, pregnancy, medicamentous immune-suppression, untreated HIV disease, agranulocytosis, active cancer), American Society of Anesthesiologists’ (ASA) score
- *Surgery and infection data*: number and type of surgeries for the actual problem, agent, dose and duration of pre-surgical antibiotic therapy, local anti-infective agents, therapeutic antibiotics, pathogens and numbers of positive cultures
- *Outcomes*: wound problems, presence and duration of vacuum-assisted negative pressure therapy, all adverse events, all nosocomial infections during and/or after hospitalization, date and reasons for re-hospitalization and re-treatment, follow-up data, fatalities for any reason

**Fig. 3:**
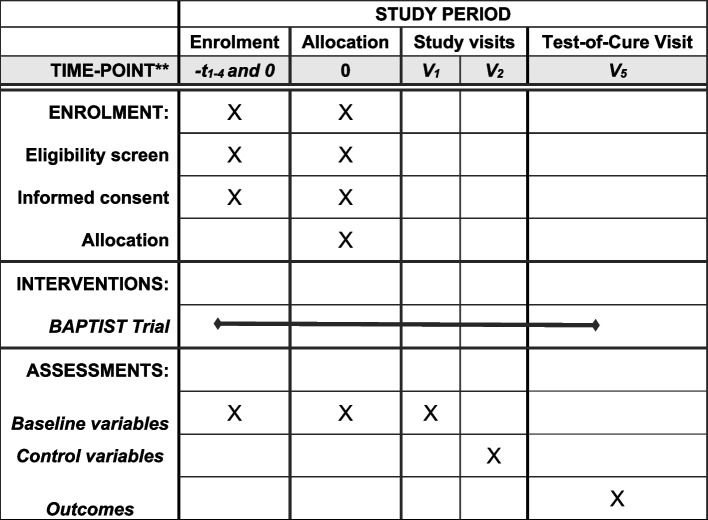
SPIRIT flowchart of enrolments and assessments during the trials. Visit times related to the Allocation (Inclusion) day: V_1_ = Day =, start of therapy, V_2_ = Day 14 (+/- 3 days; end-of-treatment visit), V_3_ = Day 42 (+/- 14 days; test-of-cure visit). Baseline variables: age, sex, known immune-suppression (diabetes mellitus, renal dialysis, cirrhosis, pregnancy, medicamentous immune-suppression, untreated HIV disease, agranulocytosis, active cancer), American Society of Anesthesiologists’ (ASA)-Score Surgery specific baseline data: number and type of surgeries for the actual problem, agent, dose and duration of pre-surgical antibiotic therapy, local antibiotics used in the bone, cell count (if any), initial serum CRP level, presence of initial bacteremia. Anatomical localization of surgery, type of surgery, microbiological results, histology (facultative). Control and outcome variables: number of surgeries to treat infection, total duration of antibiotic therapy, duration, agent and dose of intravenous and oral antibiotic therapy, wound healing problems, presence and duration of vacuum-assisted negative pressure therapy, adverse events, clinical or and microbiological recurrence, date and reasons for re-hospitalization and re-treatment, follow-up data, fatalities. Administrative data: total hospitalization length, BioBanking of infected tissues at the Balgrist Campus *The visits are all standard. The data is collected from the medical record

After surgery, the study participants will be followed up for a minimum of 12 months in case of implant and bone surgery or for 6 weeks for soft tissue surgery. The study team will equally review all medical charts to seek for unscheduled visits. During the routine study visits, we assess the history, adverse events, and the surgical status (Fig. 3).

- Visit 1—enrollment (day 1)
- End of treatment (EOT) (visit 2; end of microbiological cultures)—day 14 (± 3 days)
- Test of cure (TOC) (visit 3; surgical control after hospitalization)—day 42 (± 14 days)
- Follow-up (surgical control or by telephone) for implant surgeries—1 year (± 2 months)

### Procedures at each visit

#### Enrolment visit (day 1)

At enrollment (visit 1), the patient will be randomized into the investigational or the control prophylaxis group. The anesthetist (or the nurses on the ward) administers the prophylaxis. The study nurse collects all data. In case of (presumed) infection, the surgeons sample at least three intraoperative tissues for microbiological cultures, histology if facultative. Additionally, we usually request consent for the review of participants’ medical records and for the collection of blood and tissue samples to diagnose infection. Likewise, we concomitantly might collect and store biological specimens for genetic or molecular analyses in future (ancillary) studies.

#### Visit 2 (end of treatment)

The EOT visit corresponds to the termination of the microbiological cultures at the Laboratory for Bacteriology (IMM) in Zurich (incubated routinely during 14 days for orthopedic infections). The study nurse collects the study-specific data from the Bacterial Laboratory and from the medical and nursing files. If the patient is still hospitalized and during the entire hospitalization, the study nurse and the clinicians prospectively collect all relevant medical data in the medical and study-specific files.

#### Visit 3 (test of cure)

The TOC visit corresponds to the usual outpatient surgical control at 6 weeks post-intervention (1 year if case of implant and bone surgery). Besides the clinical controls with eventual facultative radiological, microbiological, and blood exams for clinical reasons, the clinicians and study investigators specifically ask for eventual adverse events. Additionally, the team screens all files for unscheduled microbiological results of all samples taken.

#### Follow-up for surgeries with implants in place

Usually, we see patient with implant-related and bone surgery for a surgical control after 1 year. If this is not the case for various reasons, the study nurse, or the investigators, might make a phone call to the patient for information regarding the study outcomes.

### Risks for the participants

All patients can have adverse events related to the surgery, anesthesia, and antibiotic administrations that, however, are mostly related to the surgery itself. One theoretical risk could be a higher incidence of acute nephrotoxicity, immunologic problems [19], pseudomembranous colitis, or an increase in the proportion of (multidrug)-resistant bacterial body carriage in the broad-spectrum arm [20]. We usually see such antibiotic-related adverse events during a broad-spectrum antibiotic therapy and not after a single-shot prophylaxis.

### Allocation

After written informed consent, we will randomize all participants alternately with a 1:1 ratio according to a predefined sequence. The first patient starts with the broad-prophylaxis. Concretely, the sponsor-investigator (or his replacement) obtains the signature and confirms the randomization sequence with the study nurse. Practically, the person informing and the person randomizing are different. The surgeons and the anesthesiologists cannot influence the randomization and will be informed immediately before surgery. The list of the alternate randomization sequence is on an internal computer only accessible by the investigator team.

We chose an alternate randomization method because of organizational reasons. Different teams, at any time and for any reason, regularly change the operation planning on a central website. Frequently, the planning of the early morning is not that of the afternoon. Regarding our trial, the randomization arm remains fixed to the patient. This alternating scheme will not change in cases of drop-outs, postponing of surgeries, re-scheduling of the operations, or in case of re-interventions (e.g., for hematoma or fracture). Hence, a patient randomized for a given surgery will remain in the same arm independently of the number of re-operations. If the operation order is changed on that day, the allocation remains the same. Only if a patient is re-operated for a new indication at a different body site during the 2-year study period, he/she is re-randomized and may become allocated in a opposite arm.

### Participant timetable

For the BAPTIST Trial, we probably need 24 months, starting in 25 October 2022 (Table 3).

**Table 3:** Time table of the BAPTIST Trial

**Timetable**	**Activity (year)**		**2022**			**2023**			
		**P**	**S**	**A**	**W**	**P**	**S**	**A**	**W**
P = Spring S = Summer A = Autumn W = Winter	Preparations								
	Clinical study								
	Database								
	Interim analyses								
	Final analyses								
	Writing of paper								

### Monitoring and audits

The Unit for Clinical and Applied Research (UCAR) will assign an independent monitor (with experience in prospective-randomized trials). The monitor verifies all, or a part of the CRFs, data and written informed consents. The first visit will occur prior to the start, the second during the interim analyses, and the last visit at the study end (Table 4). A quality assurance audit/inspection of this study may be conducted by the competent authority. The auditor/inspectors have access to all medical records, the investigator’s study-related files and correspondence, and the informed consent form. The investigator will allow the persons being responsible for the audit to have access to the source data/documents and to answer any questions. All involved parties will keep the patient data strictly confidential.

**Table 4:** Monitoring plan

**Study period**	**Time**	**Monitoring activities**
Before study	Autumn 2022	Monitoring will be informed about study conduct concerning data sampling and safety reporting. Monitor controls if • Documents are approved • Documents are at site • Investigators are familiar with study protocol and safety reporting • Investigators know their duties and responsibilities
Interim analysis	Autumn 2023	All subjects: Control for existence and informed consent First trial participant and at least 10% of trial participants recruited at the time of the visit, as far as available: eligibility, primary endpoint, (serious) adverse events
Study end	December 2024	Control for completeness of source data

## Statistical analyses, sample size calculations, and recruitment potential

### Sample size calculations

The daily prevalence of antibiotic use in the Balgrist is approximatively 20% (range, 15–25%). Important postoperative wound problems occur in up to 5–10% of all orthopedic interventions in high-risk patients [11]. The annual number of oncologic interventions is 120–150 surgeries. The proportion of selection of resistant bacteria is 10% [3]. We perform a superiority trial with a 10% margin and a power of 90% in favor of the broad-spectrum prophylaxis. With event-free surgeries to 95% in the broad-spectrum [1, 11] versus 85% in the standard arm [2, 3], we need 2 × 207 surgery episodes among the selected patient groups. At the Balgrist, we perform at least 6000 surgical operations per year. With a very conservative estimation, we see 300 surgical episodes each year (5%) that meet our inclusion criteria. Of note, we calculate all our estimations specifically for event-free orthopedic surgeries in tertiary centers in Switzerland and among a particular multimorbid population [1–3, 11].

### Statistical analyses

We first perform all analyses for the entire study population and stratify in a second step upon the presence of an infected implant and the orthopedic specialty. We use descriptive statistics and group comparisons using the Pearson *χ*^2^ test, the Fisher exact test, or the Wilcoxon rank-sum test, as appropriate. We also perform multivariate analysis using a Cox regression model [21]. Variables with a *p* value ≤ 0.2 in univariate analysis are included in a stepwise forward selection process for multivariate analysis. Key variables are checked for co-linearity and interaction. The number of variables in the final model is limited to the ratio of 1 variable to 5 to 8 outcome events [21]. If the BAPTIST Trial expands towards a multicenter study, or if there are more than 10% of all patients participating several times, we will add a cluster-specific analysis. The intent-to-treat (ITT) population will consist of all randomized patients. The per-protocol (PP) population will consist of all randomized patients who complete the study without important deviations from the protocol.

### Interim analyses and early termination

We perform one interim analyses after 1 year, following the inclusion of the first patient. If the differences in the primary outcomes between the broad and standard prophylaxes are important, or statistically significant, the independent data monitoring committee will decide upon the interruption or termination of the study and has the right to call on an additional interim analysis. If the group comparison analysis is not sufficiently meaningful, we consider the trial not being able to demonstrate the results, and the recruitment is no more ethical.

### Handling of missing data and drop-outs

Important missing data will lead to patients’ dropout of the study. However, we do not expect many missing data, because the intervention only consists of a prophylaxis, and all the data are routinely enregistered electronically for all surgical interventions, independently of this trial. Hence, we renounce on statistical imputations of missing data. Dropouts will be reported in the final publications, and their data will be archived for 10 years after study termination.

## Ethical and regulatory aspects

### Study registration

The study is registered in the Swiss Federal Complementary Database (BASEC 2022-00800) and in the international registry ClinicalTrials.gov (Number NCT05502380) in line with the requirements of the World Health Trial Registration Data Set. Supplementary file 1 is the original protocol. Supplementary file 2 is the checklist of for this publication.

### Categorization of this study, safety reports, and eventual amendments

This study only makes use of prophylactic antibiotic agents that are already authorized in Switzerland. The indication and the dosage are used in accordance with the prescribing information and international guidelines. The study protocol will not be changed or amended without prior sponsor’s and ethical committee’s approval. Premature interruption is reported within 30 days. The regular end of the study is reported to the ethical committee within 90 days and the final study report within 1 year. The ethical committee and authorities will receive annual safety reports and are informed about the study end. The study will be carried out in accordance with the Declaration of Helsinki, the guidelines of Good Clinical Practice (GCP), and the Swiss regulatory authority’s requirements. To communicate important modifications (e.g., changes to eligibility criteria, outcomes, analyses) to relevant parties (e.g., investigators, authorities, trial participants, registries, regulators), the sponsor and the study nurse use official channels (https://swissethics.ch/basec). This communication is via secured e-mail. Previously, they might discuss with the independent data monitoring committee.

### Patient information and early termination of the study

The investigators will inform potential participants about the study, its voluntary nature, procedures involved, expected duration, potential risks and benefits, and any potential discomfort. All participants will be provided an information sheet and informed consent form. Supplementary file 3 is a model consent form in English language. The investigators uphold the principle of the participant’s right to privacy and that they shall comply with applicable privacy laws. Subject confidentiality will be further ensured by code numbers corresponding to the computer files. For verification, the ethics committee and regulatory authorities may require access to medical records, including the medical history.

The sponsor may terminate the study prematurely in certain circumstances, e.g., ethical concerns, insufficient recruitment, when the safety of the participants is at risk, respectively, alterations in accepted clinical practice making the continuation unwise, early evidence of benefit or harm of the experimental intervention. All patients are free to withdraw from participation in this study at any time, for any reason, and without prejudice. The reason for withdrawal should be documented wherever possible. The withdrawal will not affect the actual medical assistance or future treatments. On rare occasions, the investigators may terminate a patient’s participation to protect his/her best interest. After study termination, the evaluations required at the next scheduled clinical visits will remain.

## Safety

All surgeries will be performed in the participation of an experienced surgeon. The antibiotic prophylaxis is ordered and supervised by anesthetists, internists, the pharmacy, and by infectious diseases physicians with experience in orthopedic infections.

### Definition and assessment of (serious) adverse events and other safety-related events

An adverse event (AE) is any medical occurrence in a study participant, which does not necessarily have a causal relationship with the study procedure. A serious adverse event (SAE) is classified as any untoward medical occurrence that results in death, hospitalization, or a significant prolongation of hospitalization, or persistent or significant disability or is life-threatening. The investigators make a causality assessment. All SAEs are reported within 24 h to the sponsor-investigator. SAEs resulting in death are reported to the ethics committee within 7 days. The sponsor-investigator will report the safety signals within 7 days to the local ethics committee.

### Data handling and record keeping/archiving

Data is only saved, and stored, using the secured software REDCap®. Data can only be accessed by defined persons that are investigators. An electronic case report form is generated for every participant. All data will be recorded by study nurses. The ID numbers are assigned by the REDCap® system. Corrections can only be made by authorized persons.

### Analysis and archiving

For data analysis, subject-related data from REDCap® will be exported and analyzed in a statistic software (SPSS™ and/or STATA™). All health-related data will be archived in the REDCap®. Before data export, all patient identifiers are removed. All data will be stored for a minimum of 10 years. Collection, disclosure, and storage of data is carried out in accordance with the Swiss data protection regulations and the Human Research Act.

## Discussion

The BAPTIST Trial shall demonstrate the superiority of a perioperative broad-spectrum antibiotic prophylaxis targeting against most Gram-positive and Gram-negative pathogens. However, it only investigates an adult patient population with a high risk for “prophylaxis-resistant” nosocomial infections (including SSIs), namely for patients undergoing tumor surgery [8], with open fractures [10], body colonization with multi-resistant bacteria [22], wound revisions [11], and surgeries under current antibiotic therapies (e.g., second looks of infection debridement, fracture repair with concomitant pneumonia, diabetic foot surgery during an ongoing antibiotic treatment, etc.) [3].

We exclude “standard” patients, for whom a broad-spectrum prophylaxis is very likely to fail to show benefit. Various research groups already compared a narrow-spectrum antibiotic prophylaxis to a broader one. For example, McMurtrie et al. compared cefazolin (or clindamycin) with piperacillin-tazobactam. The latter was four times more expensive but not better in reducing the infection risk [23] in open fractures. Saveli et al. performed a prospective trial in open fractures randomizing cefazolin versus a combination of cefazolin and vancomycin. The SSI risks were 15% vs. 19% [24]. Another research group retrospectively compared the efficacy of carbapenems with cefuroxime in open fractures. Carbapenems did not prevent more [25]. Redfern et al. compared cefazolin plus gentamicin versus piperacillin-tazobactam and saw similar SSI risks [26]. We also renounce on investigating the duration of prophylaxis. No data advocate that a prolonged prophylaxis reduces the SSI risk in orthopedic surgery, including in high-risk patients [1, 5].

Similarly, we know very few about non-immunologic AE related to antibiotic prophylaxes. It might be enhanced in combination prophylaxes. For example, infectious diseases experts in Boston published that, across all types of surgeries, the risk of an acute kidney injury was increased in a combined prophylaxis arm compared to a single agent (vancomycin plus a beta-lactam agent 2971/12,508 [23.8%] versus 1058/5089 [20.8%] for vancomycin alone versus 7314/52,504 [13.9%] for a beta-lactam alone) [27]. Further studies are needed. We hope our study will contribute to this lack of knowledge.

Moreover, regarding an important secondary objective, operated patients reveal more healthcare-associated infections than patients hospitalized on medical wards [28]. The SSI burden is additional to a similar same background of other bacterial nosocomial infections such as respirators tract and urinary tract of bloodstream infections [28]. Theoretically, the surgical prophylaxis might also influence the antibiotic susceptibility and the incidence of other nosocomial, non-SSI, entities, especially in urinary tract infections [29].

As a “control group,” we chose multimorbid patients undergoing orthopedic spine surgery. The reason is simple. Our circumvent surgery population comes closest to the patient population of interest in terms of co-morbidities, ASA scores, immune suppression, and, equally, the microbiology of SSI. Many scientific reports revealing the species of Gram-negative SSI in adult spine surgery report a high proportion of multi-resistant, non-fermenting rods [30, 31] that are naturally resistant to cefuroxime and maybe selected by the use of vancomycin powder [32] that the surgeons administer before wound closure at the end of the intervention.

Concerning the arbitrary spectrum of the broader prophylaxis, we choose the combination of vancomycin and gentamicin for several reasons. Both are not expensive and well tolerated. Vancomycin is already a well-established prophylactic drug in practically all surgical disciplines [1, 18] and covers almost all Gram-positive pathogens. In Switzerland, gentamicin remains susceptible to most Gram-negative pathogens, including non-fermenters and Gram-positive pathogens. Gentamicin is widely used in orthopedic surgery as a local agent for the treatment of local infections, including for wound and ulcer infections [15–17]. Theoretically, there would be other alternative agents to choose for a broad-spectrum coverage such as carbapenems. However, due to the increase of carbapenem-resistance all over the world [33, 34] and programs against their misuse [35], we renounce on carbapenems for this study.

### Limitations

We do not expect major difficulties performing the BAPTIST Trial, provided that patients and surgeons agree to participate. An important limitation is the lack of control for the surgical skills and techniques, which are paramount in the prevention of [36] but remain very difficult to analyze and are practically impossible to adjust for in a multi-variate model. On the other hand, we are a teaching hospital for orthopedic surgery with academic interest and a high volume of different types of interventions, which might help to reduce potential surgical biases as much as possible. Moreover, we perform a randomized-controlled trial with a large sample size, hoping to reduce possible biases by the randomization process. Secondly, patients who are continued to be treated outside of our hospital may have been lost or have their treatment changed. However, our center is the largest public hospital for orthopedic surgery in the region, making it again unlikely to be a major bias. Thirdly, our investigation targets all possible situations with a risk of (multidrug-resistant) SSI. We principally explore a concept of an (arbitrary) broad-spectrum prophylaxis in selected situations. In this light, the trial rather investigates a proof of concept and not specific prophylactic regimens for a given patient population or the type of orthopedic surgery, which may vary substantially among many orthopedic centers in the world. Fourthly, we perform our trial in Zurich (Central Europe). Here, the prevalence of multidrug-resistant pathogens is small in comparison to many places in the world. It could be that the results of our trial could be different in settings where this prevalence would be, let us say, 5–10 times higher [37].

In conclusion, we are confident to finish the BAPTIST Trials, to reveal some answers to some frequent questions regarding an optimized perioperative antibiotic prophylaxis in selected patients with a high risk for (prophylaxis-resistant) SSIs and other nosocomial infections [28].

### Supplementary Information

**Additional file 1: Supplementary file 1.** Clinical study protocol.

**Additional file 2: Supplementary file 2.** SPIRIT 2013 checklist.

**Additional file 3: Supplementary file 3.** Model consent form.

## Data Availability

We may provide anonymized key elements of the datasets upon reasonable scientific request.
