# Supplementary material for: Broad-spectrum antibiotic prophylaxis in tumor and infected orthopedic surgery—the prospective-randomized, microbiologist-blinded, stratified, superiority trials: BAPTIST Trials
Source: Trials. 2024 Jan 19;25:69. doi: 10.1186/s13063-023-07605-5 (PMC10799415; doi:10.1186/s13063-023-07605-5)
Supplement: Supplementary file 1 — Additional file 1: Supplementary file 1. Clinical study protocol. [file 13063_2023_7605_MOESM1_ESM.pdf]

Clinical Study Protocol

Broad-spectrum Antibiotic Prophylaxis in Tumor and Infected Orthopedic Surgery - the prospective-randomized, microbiologist-blinded, stratified, superiority Trials - BAPTIST trials

Study Type:	Clinical trial with investigational drug
Study Categorisation:	Risk category A
Study Registration:	Clinicaltrials.gov und SNCTP
Principal Sponsor:	Prof. Dr. med. Mazda FARSHAD<br>Medical Director<br>Chief Department of Orthopedic Surgery<br>Balgrist University Hospital<br>Forchstrasse 340<br>8008 Zürich<br>mazda.farshad@balgrist.ch
Principal Investigator:	Prof. Dr. med. Ilker Uçkay<br>Head Infectiology<br>Balgrist University Hospital<br>Forchstrasse 340<br>8008 Zürich<br>044 386 1111<br>ilker.uckay@balgrist.ch
Investigational Products:	None; antibiotics freely available on the Swiss market
Protocol Version and Date:	Version 1.0, 19.04.2022

CONFIDENTIAL

The information contained in this document is confidential and the property of the department of orthopaedics and infectiology of the Balgrist University Hospital. The information may not - in full or in part - be transmitted, reproduced, published, or disclosed to others than the applicable Competent Ethics Committee(s) and Regulatory Authority(ies) without prior written authorisation from Balgrist University Hospital, except to the extent necessary to obtain informed consent from those who will participate in the study.

Der Balgrist

SIGNATURE PAGE

Study number

Study title

"Broad-spectrum Antibiotic Prophylaxis in Tumour and Infected Orthopaedic Surgery - the prospective-randomized, microbiologist-blinded, stratified, superiority Trials - BAPTIST trials

The Sponsor-Investigators and trial statistician have approved the protocol version 1.0, 19.04.2022, and confirm hereby to conduct the study according to the protocol, current version of the World Medical Association Declaration of Helsinki, ICH-GCP guidelines or ISO 14155 norm if applicable and the local legally applicable requirements.

Sponsor-Investigator: Prof. Dr. med. Mazda Farshad

Zürich, 19.04.2022	
Place/Date	Signature

Local Principal Investigator:

I have read and understood this trial protocol and agree to conduct the trial as set out in this study protocol, the current version of the World Medical Association Declaration of Helsinki, ICH-GCP guidelines or ISO 14155 norm and the local legally applicable requirements.

Site:	Balgrist University Hospital<br>Forchstrasse 340<br>8008 Zürich<br>Switzerland
Principal Investigator:	Prof. Dr. med. Ilker Uçkay

Zürich, 19.04.2022	
Place/Date	Signature

Der Balgrist

TABLE OF CONTENTS

SIGNATURE PAGE 2
TABLE OF CONTENTS 3
STUDY SYNOPSIS 6-10
ABBREVIATIONS 11
STUDY SCHEDULE ....
1. STUDY ADMINISTRATIVE STRUCTURE 12
1.1 Sponsor-Investigator 12
1.3 Statistician ("Biostatistician") 12
1.4 Laboratory 12
1.5 Monitoring institution 12
1.6 Data Safety Monitoring Committee 12
1.7 Any other relevant Committee, Person, Organisation, Institution 12
2. ETHICAL AND REGULATORY ASPECTS 13
2.1 Study registration 13
2.2 Categorisation of study 13
2.3 Competent Ethics Committee (CEC) 13
2.4 Competent Authorities (CA) 13
2. 5 Ethical Conduct of the Study 13
2.6 Declaration of interest 13
2.7 Patient Information and Informed Consent 14
2.8 Participant privacy and confidentiality 14
2.9 Early termination of the study 15
2.10 Protocol amendments 15
3. BACKGROUND AND RATIONALE 15
3.1 Background Rationale 15
3.2.1 Investigational agents 17-17
3.2.2 Definitions of infections / colonizations 17
3.4 Clinical Evidence to Date 17
3.5 Dose Rationale 17
3.6 Explanation for choice of comparator 17
3.7 Risks/Benefits of the study and of BioBanking 17
3.8 Justification of choice of study population 18
4. STUDY OBJECTIVES 18
5. STUDY OUTCOMES 18
6. STUDY DESIGN 19
6.1 General study design and justification of design 19
6.1.3 Study duration 19
6.2 Methods of minimizing bias 20

6.2.1 Method of assignment to treatment/intervention (randomization, stratification) 20
6.2.2 Blinding procedures 20
6.3 Unblinding Procedures 20
7. STUDY POPULATION 20
7.1 Eligibility criteria 20
7.2 Recruitment and screening 21
7.3 Assignment to study groups 21
7.4 Criteria for withdrawal / discontinuation of participants 21
8. STUDY INTERVENTION 22
8.1 Investigational Products (treatment / medical device) 22
8.1.1 Intervention treatment 22
8.1.2 Control Comparator 22
8.1.3 Packaging, Labelling and Supply (re-supply) 22
8.1.4 Storage Conditions 22
8.2 Administration of experimental and control interventions 22
8.2.1 Experimental Intervention 22
8.2.2 Control Intervention 22
8.3 Dose modifications 22
8.4 Compliance with study intervention 22
8.5 Data Collection and Follow-up for withdrawn participants 23
8.6 Trial specific preventive measures 23
8.7 Concomitant Interventions (treatments) 23
8.8 Study Drug Accountability 23
8.9 Return or Destruction of Study Drug 23
9. STUDY ASSESSMENTS 23
9.1 Table of study procedures and assessments 23
9.2 Assessment of outcomes 24
9.2.1 Assessment of primary outcome 24
9.2.3 Assessment of other outcomes of interest 24
9.2.4 Assessment of safety outcomes 24
9.2.5 Assessments in participants who prematurely stop the study 24
9.3 Procedures at each visit for both prospective-randomized studies 25
9.3.1 Screening/Pre-procedure assessment/Visit 1 25
9.3.2 Visit 2, 3, 4, and 5 (End of Treatment) 25
9.3.3 Visit 6 (Test of Cure) 25
9.3.4 Early Termination of Study Patients 26
10. SAFETY 26
10.1 Drug studies 26

10.1.1 Treatment by specialists at UKB 26
10.1.2 Definition and assessment of (serious) adverse events 26
10.1.3 Reporting of serious adverse events (SAE) 27
10.1.4 Follow up of (Serious) Adverse Events 28
11. STATISTICAL METHODS 29
11.1 Main hypotheses 29
11.2 Determination of Sample Size 29
11.3 Planned Analyses 29
11.3.1 Interim analyses and early termination of the RCT 29
11.3.2 Final analyses 29
11.4 Handling of missing data and drop-outs 30
12. QUALITY ASSURANCE AND CONTROL 30
12.1 Data handling and record keeping / archiving 30
12.1.1 Case Report Forms 31
12.1.2 Specification of source documents 31
12.13 Record keeping / archiving 31
12.2 Data Management System, access and back-up 32
12.2.1 Analysis and archiving 32
12.3 Monitoring 32
12.4 Audits and Inspections 33
12.5 Confidentiality, Data Protection 33
12.6 Storage of biological material and related health data 33
13. PUBLICATION AND DISSEMINATION POLICY 33
14. FUNDING AND SUPPORT 33
15. INSURANCE 34
16. REFERENCES 34

SYNOPSIS

**Sponsor-Investigator(s)**	Prof. Dr. med. Mazda Farshad, Medical Director, Balgrist University Hospital
**Principal Investigator(s)**	Prof. Dr. med. Ilker Uçkay, Head of Clinical Research UCAR (Unit of Clinical and Applied Research)
**Study Title:**	**B**road-spectrum **A**ntibiotic **P**rophylaxis in **T**umor and **I**nfected Orthopedic **Su**rgery – the prospective-randomized, microbiologist-blinded, stratified, superiority **T**rials - **BAPTIST** trials
	The perioperative antibiotic prophylaxis is evidence-based in orthopedic surgery. While its duration ranges from a single dose to three doses throughout the world, the choice of the prophylactic agents is undisputed. Worldwide, the surgeons use 1st or 2nd-generation cephalosporins (or vancomycin in some cases).<br>However, there are particular clinical situations with a high risk of antibiotic-resistant surgical site infections (SSI); independently of the duration of administered prophylaxis. These resistant SSI's occur in contaminated wounds, or during surgery under current therapeutic antibiotics, and base on "***selection***" by antibiotics used for therapy or prophylaxis.
**Short Title / Study ID:**	The **BAPTIST** trials
**Protocol Version and Date:**	Version 1.0; 19. April 2022
**Trial registration:**	Swiss National Clinical Trials Portal (SNCTP) and the international trial registry ClinicalTrials.gov (www.clinicaltrials.gov). Publication of the protocol in "Trials".
**Study category and Rationale**	Category A: All prophylactic antibiotics used are authorized by *Swissmedic* already as therapeutic agents.
**Clinical Phase:**	Not applicable; the prophylactic antibiotics are used according to the prescribing information.

**Background and Rationale:**	The perioperative antibiotic prophylaxis is evidence-based for the majority of orthopedic surgeries[1]. While the debate of its duration and timing (single-dose versus triple dose; before or after the intraoperative microbiological sampling) may continue, no clinicians doubt on the efficiency of the recommended prophylactic agents; that are mostly 1st or 2nd -generation cephalosporins[1], co-amoxiclav or other exceptional agents in cases of (pseudo)-allergy[1]. However, and traditionally, up to the half of all detected pathogens of orthopedic SSI's are not covered by the prior prophylactic regimen[2-5]: e.g. SSIs due to methicillin-resistant cocci[5] or non-fermenting Gram-negative rods in orthopedic surgery[4].<br><br>Additionally, orthopedic surgeons operating selected patient populations (neoplasms[6,7], open fractures[8], postoperative wound dehiscence[9], diabetic foot infections[2,10] or already infected body sites[2,11]) experience a high risk of prophylactic-resistant pathogens, or pathogens resistant to current therapeutic antibiotics regimens. At least 10% of all new intraoperative tissue samples, during iterative surgical debridement, yield (new) pathogens unknown to the clinicians[2]. This is due to selection by prophylactic or therapeutic antibiotics, which only kill the previously detected pathogens, but left over newly introduced contaminants, remnant parts of partially-diagnosed polymicrobial infections; ultimately leading to a new SSIs occurring during therapy for the first infection at the orthopedic site. This selection is unpredictable involving both Gram-positive skin pathogens as well as (multi) resistant Gram-negative rods[2]. From a microbiological point of view, only a maximal Gram-negative coverage, alongside with a large Gram-negative coverage, would cover these selections.<br><br>The literature is in-existing how to prevent these selections. Most clinicians just continue with the standard prophylactic recommendation, or the current therapeutic antibiotic regimen. Theoretically, we cannot exclude that these selected patient populations eventually might profit from a broad-spectrum prophylaxis.

**Study Conduct**	The BAPTIST trials only concern the perioperative antibiotic prophylaxis in selected situations of orthopedic surgery: tumor surgery, debridement for postoperative dehiscent wounds, debridement under antibiotics, open fractures, skin colonization with multidrug-resistant bacteria, plus, as a control; spine surgery in selected multimorbid patients. We alternately randomize the standard prophylaxis (or by continuing the current antibiotic treatment) to the additional broad-spectrum single-shot of vancomycin 1g IV & single-shot of gentamicin 5 mg/kg intravenously; before an eventual intraoperative sampling. End-of-Treatment (EOT) and / or Test-of-Cure (TOC) occur latest at the 6-week's surgical control visit. The rest of the hospital stay, treatment, the use of negative-pressure vacuum therapy, other interventions, local antibiotic therapies; therapies or procedure are at the discretion of the treating clinicians.<br><br>We will randomize surgical interventions defined by the inclusion criteria in a prospective-alternating scheme (1:1) according to the scheduled position in the operating theatres. The anesthetists (or the nurses at the hospitalization units) will administer the standard prophylaxis (or the therapeutic antibiotics) alone, or with the addition of the single-shot broad-spectrum prophylaxis regimen composed of vancomycin and gentamicin. In case of clinical suspicion of infection or massive contamination, the surgeons will perform at least three microbiological intraoperative tissue samples. Each surgery counts as an independent event. If a patient is debrided several times, he / she can have different prophylaxis regimens during each of the interventions. After the prophylactic regimen, the clinicians are free to continue with a targeted or empirical therapeutic antibiotic regimen. The antibiotic therapy *per se* is not an objective of this current trials.<br><br>The treatment period includes the following daily study visits:<br>• Visit 1 - Enrollment (Day 1)<br>• EOT (end of microbiological cultures) - Day 14 (+/- 3 days)<br>• TOC (clinical surgical control) - Day 42 (+/- 14 days)<br>• Follow-up (telephone) for implant-related surgery - 1 year (+/- 2 months)
**Objective(s):**	We determine the microbiology of the deep surgical site, or its selection under current antibiotic therapy, in pre-defined complex orthopaedic populations. We are interested if a broad-spectrum antibiotic prophylaxis may significantly reduce the nature and extent of eventual new antibiotic-resistant pathogens.

**Outcome(s):**	Primary outcome<br>- Remission (and inversely SSI) at 6 weeks for surgeries without implant and 1 year for surgeries with implant<br><br>Secondary outcomes:<br>- Risk of (antibiotic-resistant) pathogens in the deep surgical site of the study patients; in relation to the prior antibiotic prophylaxis or the current therapeutic antibiotic therapy within 6 weeks<br>- Revision surgery for non-infections reasons within 6 weeks<br>- Change of antibiotic therapy based on intraoperative findings<br>- Spectrum, adverse events and costs of therapeutic antibiotics (if any)<br>- Incidence of non-SSI infections within 6 weeks (e.g. urine infections)<br>- Persistence of skin and body colonization with Gram-negative multi-resistant bacteria at 4-6 weeks (if any samples)
**Study design**	Prospective-randomized, microbiologist-blinded, stratified, superiority trials
**Inclusion / Exclusion criteria:**	Inclusion criteria:<br>• Surgery under current or recent therapeutic antibiotics (antibiotic-free window <14 days and during more than 4 days)<br>• Surgery for open fractures; including 2nd and 3rd looks<br>• Potentially contaminated wound revision in the operating theatre<br>• Tumor (oncologic) orthopedic surgery with prior radiotherapy and / or bone involvement<br>• Spine surgery with ASA-Score ≥ 3 points, sacral involvement, and or revision surgery<br>• Known skin colonization with multidrug-resistant Gram-negative bacteria defined by infection control undergoing surgery<br><br>Exclusion criteria:<br>• Surgery without intraoperative microbiological samples<br>• Allergy or major intolerance to vancomycin and/or gentamicin<br>• Anticipated clinical follow-up of less than 6 weeks after inclusion<br>• Pregnant or breastfeeding women<br>• Known carriage of resistant Gram-negative bacteria only in urine or anal
**Definitions**	**Standard prophylaxis:**<br>1 - 3rd generation cephalosporins, co-amoxiclav, clindamycin<br>**Broad-spectrum prophylaxis**:<br>vancomycin 1 g & gentamicin 5 mg/kg.<br>**Broad-spectrum therapy:**<br>4 - 5th generation cephalosporins, quinolones, carbapenems, vancomycin, daptomycin, linezolid, aminoglycosides, colistin, metronidazole

<table>
<tr><td>Measure-ments and procedures</td><td>We assess:
– Patient's characteristics (age, sex, body mass index, renal insufficiency) and: antibiotic prophylaxis and microbiology, the indication of surgery, presence of osteosynthesis material, local antibiotic or antiseptic use, diabetes and other immune suppression states, wound discharge, seroma, hematoma, surgical site infection, all postoperative infections, number of surgical lavages and debridement, adverse events of prophylactic antibiotic therapy and duration, length of hospital stay, duration of VAC / PICO use[12], body and skin colonization with multi-resistant Gram-negative bacteria.</td></tr>
<tr><td>Study Product</td><td>None. All prophylactic antibiotics are freely available on the Swiss market.</td></tr>
<tr><td>Timetable

P = Spring
S = Summer
A = Autumn
W = Winter</td><td>
<table>
<tr><th>Activity (year)</th><th colspan="4">2022</th><th colspan="4">2023</th></tr>
<tr><th></th><th>P</th><th>S</th><th>A</th><th>W</th><th>P</th><th>S</th><th>A</th><th>W</th></tr>
<tr><td>Preparations</td><td>■</td><td>■</td><td></td><td></td><td></td><td></td><td></td><td></td></tr>
<tr><td>Clinical study</td><td></td><td>■</td><td>■</td><td>■</td><td>■</td><td>■</td><td>■</td><td></td></tr>
<tr><td>Database</td><td></td><td>■</td><td>■</td><td>■</td><td>■</td><td>■</td><td>■</td><td>■</td></tr>
<tr><td>Interim analyses</td><td></td><td></td><td></td><td>■</td><td>■</td><td></td><td></td><td></td></tr>
<tr><td>Final analyses</td><td></td><td></td><td></td><td></td><td></td><td></td><td>■</td><td>■</td></tr>
<tr><td>Writing of paper</td><td></td><td></td><td></td><td></td><td></td><td></td><td>■</td><td>■</td></tr>
</table>
</td></tr>
<tr><td>Number of Participants with Rationale:</td><td>Each day, the prevalence of therapeutic antibiotic use among our hospitalized patients is approximatively 20 % (range, 15 - 25 %). Postoperative wound problems occur in up to 5 % of all interventions[8]. The number of annual oncologic interventions is 120 - 150 surgeries. The performance of the antibiotic prophylaxis to reduce SSI in orthopedic surgery is 5 – 10 %[1]. Per analogy, the performance of a broad-spectrum prophylaxis to reduce antibiotic-resistant intraoperative samples is probably 5 – 10 %. The proportion of selection of antibiotic-resistant new bacteria in is 10 %[2]. We perform a superiority RCT with a 10 % margin and a power of 90 % in favor of the broad-spectrum prophylaxis. With event-free surgeries to 95 % in the broad-spectrum versus 85 % in the standard arm, we need 2 x 207 surgery episodes among the selected patient groups. At the Balgrist, we perform at least 5000 surgical operations per year. With a very conservative estimation, we see 300 surgical episodes per year (6 %) that can be included in our RCT, and its stratifications. The study will last two years; with an interim analysis after one year. An independent advisory board might stop the RCT.</td></tr>
<tr><td>GCP Statement:</td><td>This study will be conducted in compliance with the protocol, the Declaration of Helsinki, the ICH-GCP and national legal and regulatory requirements.</td></tr>
<tr><td>Insurance</td><td>The trials are covered by the research insurance of the Balgrist.</td></tr>
</table>

Der Balgrist

ABBREVIATIONS

AE	Adverse Event
ASA	American Society of Anaesthesiologists
ASR	Annual Safety Report
CA	Competent authority
CEC	Competent Ethics Committee
CRP	Serum C-reactive protein
eCRF	Electronic case report forms
EOT	End of treatment
GCP	Good Clinical Practice
ICH-GCP	International Conference on Harmonization of Good Clinical Practice
IMM	Institut für Medizinische Mikrobiologie
NRS	Nutritional Risk Screening
NDI	Neck disability index
ODI	Oswestry low back disability index
PD	Privatdozent
REDCap	Research Electronic Data Capture
SAE	Serious Adverse Events
SNCTP	Swiss National Clinical Trials Portal
SOP	Standard Operation Procedure
SUSARs	Suspected Unexpected Serious Adverse Reactions
TOC	Test of Cure
UCAR	Unit for Clinical and Applied Research
UKB	Universitätsklinik Balgrist
VAC	Vacuum-Assisted Negative Pressure
ZLZ	Zentrallabor Zürich

1. STUDY ADMINISTRATIVE STRUCTURE

1.1 Sponsor-Investigators

Prof. Dr. med. Mazda Farshad, Surgeon in Chief and Medical Director,

Balgrist University Hospital, Forchstrasse 340, 8008 Zürich

The sponsors are responsible for trial design and management, data handling and record keeping, subject protection, quality management, financing, investigational product management and safety evaluation. He ensures oversight and designates appropriately qualified personnel. The sponsor is going to supervise data collection, management and integrity as well as analysis and interpretation.

1.2 Principal Investigators:

Prof. Dr. med. Ilker Uçkay, Head Infectiology, Head of Clinical Research UCR (Unit for Clinical and Applied Research), Balgrist University Hospital, Forchstrasse 340, 8008 Zürich

The PI's are responsible for the protocol and GCP conform conduct of the trial at the site. They delegate and supervises trial-related duties to qualified staff and ensures medical care of trial subjects. The PI ensures that randomization and informed consent procedures are followed, source and CRF records are accurate and that safety reporting requirements are met.

1.3 Statistician ("Biostatistician")

Statistical analyses will be performed by the investigators (and the biostatistician Mr. Tobias Götschi of UCAR) using SPSS and/or STATA software (Version 14). In case of necessity, other biostatisticians will be consulted.

1.4 Laboratory

Bacterial analyses are performed clinically at the IMM, in the immediate vicinity of UKB, as part of the regular analysis of the clinical course. This study does not asses serum laboratory parameters.

1.5 Monitoring institution

An internal study monitoring board is established to perform ongoing study surveillance and to perform interim analyses if appropriate. UCAR (Unit for Clinical and Applied Research); Prof. Dr. med. Ilker Uçkay, Balgrist Campus, Lengghalde 5, 8008 Zürich.

1.6 Data Safety Monitoring Committee

A data safety committee of two persons with experience in clinical research and biostatistics who are not part of the co-investigators or future authors of the scientific publication will monitor the safety of data and of the study; one interim analysis (after one year (+/- 2 months).

1.7 Any other relevant Committee, Person, Organisation, Institution

n/a

2. ETHICAL AND REGULATORY ASPECTS

The decision of the Competent Ethics Committee (CEC) concerning the conduct of the study will be made in writing to the Sponsor-Investigator before commencement of this study. The clinical study can only begin once approval from the CEC has been received. Any requirements imposed by the authorities shall be implemented.

2.1 Study registration

The study will be registered at http://www.clinicaltrials.gov and http://www.snctp.ch.

2.2 Categorization of study

This study only makes use of the medicinal products which are already authorized in Switzerland for the treatment of surgical sites infections, including with materials. The indication and the dosage are used in accordance with the prescribing information and the international guidelines. All drugs and doses in this study are commonly used treatment methods and freely available on the Swiss market since decades. There will be no placebo.

2.3 Competent Ethics Committee (CEC)

The principal investigator ensures that approval from an appropriately constituted Competent Ethics Committee (CEC) is sought for this clinical study.

The reporting duties such as all changes in research activity, all unanticipated problems involving risks to humans and planned or premature study end and the allowed time frame are respected by this study. The study protocol will not be changed without prior Sponsor and CEC approval, except when it's necessary to eliminate apparent immediate hazards to study participants.

Premature study end or interruption of the study is reported within 15 days. The regular end of the study is reported to the CEC within 90 days, the final study report shall be submitted within one year after study end. Amendments are reported according to chapter 2.10.

2.4 Competent Authorities (CA)

CA (*swissmedic*) approval is only necessary for category B and C studies. Categories A studies do not require CA approval. The CA is entitled to carry out inspections of all clinical trials. This trial is category A.

2.5 Ethical Conduct of the Study

The study will be carried out in accordance to the protocol and with principles enunciated in the current version of the Declaration of Helsinki, the guidelines of Good Clinical Practice (GCP) issued by ICH, the Swiss Law and Swiss regulatory authority's requirements. The CEC and regulatory authorities will receive annual safety and interim reports and be informed about study stop/end in agreement with local requirements.

2.6 Declaration of interest

No conflict of interest compromises the professional judgement of our investigators and the other involved people in conducting and reviewing this study. Their objectivity is not influenced in any way (e.g. independence, intellectual, financial, proprietary) or through any party.

2.7 Patient Information and Informed Consent

For the participation in the study, patients will be recruited/preselected by any of the investigators of the study. If patients match the inclusion criteria and do not meet any exclusion criterion for the study, they will be informed by one of the study investigators and study nurses / research assistants, about the study, its nature, purpose, procedures involved, expected duration, participating investigators, potential risks and benefits, any potential discomfort the study could entail, and during the clinical control visit at 4-6 weeks (and 1 year for implant-related surgery). Each participant will be informed that the participation in the study is completely voluntary and that he/she may withdraw from the study at any time and that withdrawal of consent will not affect his/her medical assistance and treatment in the future. No further screening requirements (other than the in- and exclusion criteria) exist.

All participants will be provided a participant information sheet and informed consent form describing the study and entailing sufficient information for the participant to make an informed decision about their willingness to participate in the study. The patient information sheet and the consent form will be submitted to the CEC to be reviewed and approved. The information sheet provides the possibility to read through the study concept again and enables the patient to rethink the study participation without being pressured into decision making. If the participant decides to take part in the study, he/she will be asked to date and sign the informed consent form. The potential participant will be requested to read through the consent form and the information sheet carefully and to clarify any misunderstandings before signing. Once the patient dates and sign the informed consent form, one of the investigators will also date and sign the aforementioned document. The participant will be given a copy of the signed document. The original signed informed consent form will be retained as part of the study records.

The formal consent of a participant, using the approved consent form, must be obtained before the participant is submitted to any study specific procedures.

All patients of whom intraoperative tissue and/or bone samples are collected and stored in the Bio Bank have signed the general consent „Einwilligungserklärung zur Weiterverwendung von biologischem Material und gesundheitsbezogenen Personendaten für die Forschung “, or the corresponding section of the study specific consent.

The collection of the general consents for further use of health-related personal data and biological material is a standard at UKB. It is not study-specific.

2.8 Participant privacy and confidentiality

The investigators affirm and uphold the principle of the participant's right to privacy and that they shall comply with applicable privacy laws. Especially, anonymity of the participants shall be guaranteed when presenting the data at scientific meetings or publishing them in scientific journals.

Individual subject medical information obtained as a result of this study is considered confidential and disclosure to third parties is prohibited. Subject confidentiality will be further ensured by utilising subject identification code numbers to correspond to treatment data in the computer files.

For data verification purposes, authorised representatives of the Sponsor (-Investigator), a competent authority (e.g. *Swissmedic*), or an ethics committee may require direct access to parts of the medical records relevant to the study, including participants' medical history.

2.9 Early termination of the study

The Sponsor-Investigators may terminate the study prematurely according to certain circumstances, for example:

- ethical concerns
- insufficient participant recruitment
- when the safety of the participants is doubtful or at risk, respectively
- alterations in accepted clinical practice that make the continuation of a clinical trial unwise
- early evidence of benefit or harm of the experimental intervention, e.g. based on interim analyses

2.10 Protocol amendments

Substantial amendments are only implemented after approval of the CEC.

Under emergency circumstances, deviations from the protocol to protect the rights, safety and well-being of human subjects may proceed without prior approval of the CEC. Such deviations shall be documented and reported to the CEC as soon as possible.

All non-substantial amendments are communicated to the CEC within the Annual Safety Report (ASR).

As substantial Amendments Count:

a) Changes, which affect security and health of the participants or their rights and duties.
b) Changes of the study protocol, due to new scientific findings, which affect study arrangement, study methods, objectives or statistical analysis.
c) Changes of the study location or inclusion of an additional study location.
d) Major personnel changes, such as change of the sponsor-investigators.

1. BACKGROUND AND RATIONALE

3.1 Background Rationale

The perioperative antibiotic prophylaxis is evidence-based for the majority of orthopedic surgeries[1]. While the debate of its duration and timing (single-dose versus triple dose; before or after the intraoperative microbiological sampling) may continue, no clinicians doubt on the efficiency of the recommended prophylactic agents. These agents are mostly 1st or 2nd generation cephalosporins[1], co-amoxiclav or other drugs in exceptional cases of (pseudo)-allergy[1].

However, and traditionally, up to the half of all detected pathogens of orthopedic SSI's are not covered by the prior prophylactic regimen[2-5]: e.g. SSIs due to methicillin-resistant cocci[5] or non-fermenting Gram-negative rods in orthopedic surgery[4]. Additionally, orthopedic surgeons operating selected patient populations (neoplasms[6,7], open fractures[8], postoperative wound dehiscence[9], diabetic foot infections[2,10] or already infected body sites[2,11]) experience a high risk of prophylactic-resistant pathogens, or pathogens resistant to current therapeutic antibiotics regimens. At least 10% of all new intraoperative tissue samples, during iterative surgical debridement, yield (new)

pathogens unknown to the clinicians[2]. This is due to selection by prophylactic or therapeutic antibiotics, which only kill the previously detected pathogens. In case of open wounds, the current (actual) antibiotics left over newly introduced contaminants, remnant parts of partially-diagnosed polymicrobial infections; and maybe be selective in favor of a new SSIs occurring during therapy for the first infection at the same orthopedic site.

The literature is in-existing how to prevent these selections. Most just continue with the standard recommendation, or the current therapeutic antibiotic regimen. Or they simply prolong the recommended prophylaxis duration by several days by narrow-spectrum agents hoping that the prolonged administration of a standard antibiotic prophylaxis might prevent (antibiotic-resistant) SSI. In contrast, we think that in anticipation of future antibiotic-resistant pathogens, some clinicians rather may use broad-spectrum antibiotics for a short time, instead of prolonging narrow-spectrum regimens. This praxis is equally existing and a part of the clinical case management, but needs an RCT to be investigated.

The BAPTIST trials only concern the perioperative antibiotic prophylaxis in such selected situations of orthopedic surgery: tumor surgery, debridement for postoperative dehiscent wounds, debridement under antibiotics, open fractures, skin colonization with multidrug-resistant bacteria, plus, as a control; spine surgery in selected multimorbid patients. We alternately randomize the standard prophylaxis (or continuing the current antibiotic treatment) to the additional broad-spectrum single-shot of vancomycin 1g IV & single-shot of gentamicin 5 mg/kg intravenously; before the eventual intraoperative samplings. EOT occurs at the end of microbiological incubation at 14 days. The TOC visit in the surgical clinical control. It traditionally occurs latest at the 4-6-week's surgical control visit. The rest of the hospital stay, treatment, the use of negative-pressure vacuum therapy, other interventions, local antibiotic therapies; therapies or procedure are at the discretion of the treating clinicians.

3.2 Investigational Product and Indication

Antibiotic agents used in the study

The antibiotic prophylaxis, even the broad-spectrum single-shot of vancomycin and gentamicin, is very few expensive in relation to the therapy or the surgical intervention. Moreover, vancomycin is already a well-established prophylactic agent in practically all surgical disciplines and covers almost every Gram-positive pathogen, including skin commensals, which are dominant in open wounds. Gentamicin is well tolerated as single-shots, has a very low risk profile of allergic reactions and covers most Gram-negative pathogens, including Gram-negative rods, non-fermenters and Gram-positive pathogens. Moreover, gentamicin is widely used in orthopedic surgery as a local agent for the prevention and therapy of all sorts of orthopedic infections, including wound and foot ulcer infections[14-16]. This combination of vancomycin and gentamicin covers the usual pathogens that microbiologically escape to standard narrow-spectrum prophylaxis. Theoretically, there would be alternative agents for broad-spectrum coverage such as carbapenems or related agents. However, due to the increase of carbapenem-resistant pathogens all over the world, and programs against their misuse in orthopedic surgery and diabetic foot surgery[17], we renounce on the use of carbapenem antibiotics for this study.

A further issue could be the potential of selecting resistant pathogens in the individual host. This danger has been published for prophylaxis lasting more than 48 hours, also in Switzerland[18]. Regarding single-shot prophylaxis, we ignore any scientific evaluation proofing that single-shot applications of vancomycin or gentamicin would lead to an enhanced proportion of body carriage of multi-resistant bacteria.

We will not test special doses, placebos or new indications for antibiotic therapy. Even in case of an acute, or chronic, renal insufficiency, we will not alter the dosing of the single-shot prophylaxis in congruence with international practice[1]. Only the duration of the therapy of prophylaxis will be determined. All antibiotics are already on the Swiss market and approved by *Swissmedic*. Of note, anesthesiologists and surgeon are free to administer local antibiotic or antiseptic agents, or to continue with therapeutic antibiotics, which we will record.

3.2.1 Standard antibiotic prophylaxis used in the study

The standard prophylaxis used at UKB consists of one to three intravenous doses of cefuroxime 1.5 g intravenously; or 3g if obesity; or vancomycin 1 g or clindamycin 600 mg; if allergy. For this study, we do not determine the scientific necessity of prophylaxis. All patients will receive any form of prophylaxis.

3.2.2 Definitions of orthopedic infection, spectrum of antibiotic prophylaxis and wound problems

For this RCT, we define a persistent or new infection at the surgical site (SSI) as the microbiological evidence of bacteria in at least two intraoperative tissue samples together with radiological (osteomyelitis, collections, inflammation) and/or clinical evidence of infection (pus, discharge, abscess, rubor, calor, pain, sinus tract). Histological proof is facultative for this study.

Remission of infection is the absence of clinical and/or radiological and/or laboratory signs of (former) infection after the minimal follow-up time of 30 days (or 1 year in case of implant-related surgery with implants in place)[1,13].

Wound problems are a group of non-infected wound disturbances, of which clinical entities such as seroma, hematoma or dry dehiscence predominate[9].

Standard prophylaxis: 1-3rd generation cephalosporins, clindamycin

Broad-spectrum prophylaxis: vancomycin 1 g & gentamicin 5 mg/kg.

3.4 Clinical Evidence to Date

See chapter 3.1

3.5 Dose Rationale

See chapter 3.1

3.6 Explanation for choice of comparator

See chapter 3.1

3.7 Risks/Benefits of the study

As a benefit, episodes with a broader perioperative prophylaxis might witness less SSI, re-operations and/or postoperative wound problems. If this is the case, future patients might profit from this knowledge.

All patients can witness adverse events related to surgical procedures and antibiotic prophylaxis, which however, are not necessarily specific to the study protocol. The possible adverse events related to the administration of vancomycin and gentamicin, are a Red Man syndrome, immunologic problems or a(pseudo)allergy[19]. In contrast, with only a single-shot of this broad-spectrum combination, we do not expect a pejoration of the renal insufficiency or a hearing loss that usually occur in case of prolonged administration.

Finally, the surgical and anesthesiologic procedures harbors a lot of inherent risks, which are all due to the therapy and procedure itself (and not due to the study). In this study, we also record these non-study-specific complications.

3.7.1. Pregnancy and breast-feeding

The surgeries have no specific relations to pregnant or breast-feeding women and their children. Additionally, the study population is likely not to reveal women at procreating age. However, due to antibiotic administrations, pregnant and breast-feeding women are excluded from this study. No study-specific pregnancy tests will be performed.

3.8 Justification of choice of study population

See chapter 3.1. No vulnerable participants are included.

4. STUDY OBJECTIVES

We determine the incidence of SSI and postoperative wound problems, alongside with the changing microbiology of the deep surgical site (open wound) in relation to the spectrum of initial perioperative antibiotic prophylaxis or therapy. We are interested if a broad-spectrum antibiotic prophylaxis may significantly reduce the nature and extent of adverse outcomes and of the occurrence of antibiotic-resistant pathogens.

5. STUDY OUTCOMES PRIMARY OUTCOME

Primary outcome

- Remission (and inversely SSI) at 6 weeks for surgeries without implant and 1 year for surgeries with implant

Secondary outcomes

- Risk of (antibiotic-resistant) pathogens in the deep surgical site of the study patients; in relation to the prior antibiotic prophylaxis or the current therapeutic antibiotic therapy within 6 weeks
- Revision surgery for non-infections reasons within 6 weeks
- Change of antibiotic therapy based on intraoperative findings
- Spectrum, adverse events and costs of therapeutic antibiotics (if any)
- Incidence of non-SSI infections within 6 weeks (e.g. urine infections)

- Persistence of skin and body colonization with Gram-negative multi-resistant bacteria at 4-6 weeks (if there are any samples)

6. STUDY DESIGN

6.1 General study design and justification of design

We prospectively collect only the following parameters:

Age, biological sex, weight, body mass index, antibiotic prophylaxis (therapy) and duration, intraoperative microbiology (if any), indication of surgery, osteosynthesis material, local antibiotic or antiseptic use, diabetes and other immune suppression states, duration of VAC / PICO use[12], body and skin colonization with multi-resistant Gram-negative bacteria (if any). Intraoperative bacterial microbiology (if any), wound discharge, seroma, hematoma, surgical site infection, all postoperative infections, number of surgical lavages and debridement, adverse events of prophylactic antibiotic therapy and duration. Remission, clinical and microbiological recurrences, re-operation, wound problems, adverse events of prophylactic antibiotic therapy and duration, body and skin carriage with multi-resistant Gram-negative bacteria (if any), occurrence of other, non-SSI-infections in the study period.

The prophylactic antibiotic regimen is administered according to current practice. We will randomize surgical interventions defined by the inclusion criteria in a prospective-alternating scheme (1:1) according to the scheduled position for operation. The anesthetists or the hospitalization units will administer the standard prophylaxis (or the therapeutic antibiotics) alone, or with the addition of the single-shot broad-spectrum prophylaxis regimen. In case of clinical suspicion of infection or massive contamination, the surgeons will perform at least three intraoperative tissue samples for bacteriology. Each surgery counts as an independent event. If a patient is debrided several times, he/she can have different prophylaxis regimens during each of the interventions. After the prophylactic regimen, the clinicians are free to continue with a therapeutic antibiotic regimen. The pre- or postoperative antibiotic treatment *per se* is not an objective of this current trials.

6.1.2 In case of refusal or withdrawal from the study

When a patient refuses to take part of the study, or is rejected by the investigators, his/her treatment will continue according to usual therapeutic standards and follow-ups. If a patient withdrawals his/her consent during the study period or at the TOC, his/her information and results will be deleted from further analyses.

6.1.3 Study duration

For the randomized study, we count two years; starting in winter/spring 2022 (CEC approval provided). The following Table highlights some key time events. The RCT starts at UKB, but is expansible to other centers (provided the approval of the different Ethical Committees upon amendment / submission).

Activity (year)	2022				2023			
	P	S	A	W	P	S	A	W
Preparations	■	■						
Clinical study		■	■	■	■	■	■	
Database		■	■	■	■	■	■	■
Interim analyses				■	■			
Final analyses							■	■
Writing of paper							■	■

P = spring, S = summer, A = autumn, W = winter

6.2 Methods of minimizing bias

The methods of minimising bias applied in our study are the randomization to add validity of the statistical tests used to demonstrate significance. The differences between intervention and control groups should behave like differences between two random samples from the population so that they can be compared to what would be expected in the population by chance.

6.2.1 Method of assignment to treatment/intervention (randomization, stratification)

After written informed consent has been given, participants will be randomized with a 1:1 ratio in either treatment group. Randomization is performed electronically. Patients are informed about the assignment by the treating clinicians.

6.2.2 Blinding procedures

There will be no blinding of patients, and no placebos.

6.3 Unblinding Procedures

n.a.

7. STUDY POPULATION

7.1 Eligibility criteria

Only selected orthopedic patients with complex clinical situations and an elevated risk for antibiotic-resistant SSI and wound contamination will participate. The complex spine surgery serves as a control part of surgery with closed wounds.

Inclusion criteria:

- Age ≥ 18 years
- Surgery under current or recent therapeutic antibiotics (antibiotic-free window <14 days and past antibiotic prescription during more than 4 days)
- Surgery for open fractures and wounds; including 2nd and 3rd looks

• Potentially contaminated wound revision in the operating theatre<br>• Tumor (oncologic) orthopedic surgery (only if prior radiotherapy and/or bone involvement)<br>• Spine surgery with ASA-Score >= 3 points, sacral involvement, and/or revision surgery<br>• Known skin colonization during surgery with multidrug-resistant Gram-negative bacteria, defined by the local infection control team<br><br><u>Exclusion criteria</u>:<br>• Inability to understand the study procedure for linguistic or cognitive reasons<br>• Surgery without intraoperative microbiological samples<br>• Allergy or major intolerance to vancomycin and/or gentamicin<br>• Anticipated clinical follow-up of less than 6 weeks after inclusion<br>• Pregnant or breastfeeding women<br>• Known carriage of resistant Gram-negative bacteria only in the urine or anal

7.2 Recruitment and screening

The patients are screened during the preoperative surgical and anesthesiologic assessments, and again during the morning colloquium of the orthopedic surgeons. In case all inclusion and no exclusion criteria are met, the patient will be informed about the study by one of the study investigators or a study nurse / research assistant (see chapter 2.7). Study nurses / research assistants and / or the individual surgical teams include the corresponding patients into the RCT and report it to a central study nurse / central research assistant (the PI or the Sponsor) of the study.

7.3 Assignment to study groups

Assignment to the study group (control versus intervention prophylaxis group) will be performed electronically by the central study nurse / central research assistant according to a prospective-alternating scheme (1:1); based on the scheduled position in the operating theatres.

7.4 Criteria for withdrawal / discontinuation of participants

All patients are free to withdraw from participation in this study at any time, for any reason, and without prejudice. A patient who withdraws consent by refusing to continue with study procedures/observations will be terminated from the study. The reason for withdrawal of consent should be clearly documented wherever possible. However, it is not required for patients to provide their reason.

The investigator should make every effort to address non-compliance issues and ensure that relevant study data are obtained from patients whenever possible.

To enable collection of follow-up data, the investigator may stop study treatment at any time without withdrawing the patient from the study (e.g. the patient experiences intolerable or unacceptable AEs possibly related to study treatment and where such

treatment cannot be modified within the confines of the protocol).

On rare occasions, the investigator may terminate a patient from the study to protect the patient's best interest e.g. to protect them from excessive risk or risk with a demonstrated lack of benefits (serious side-effects) or to maintain the integrity of the data (when participates are not following study procedures or may be deliberately providing false information). The investigator must explain to the participant the reasons for the termination. If a patient is withdrawn before completing the study, the reason for withdrawal will be entered in the electronic case report form (eCRF). Whenever possible and reasonable, the evaluations that are required at the next scheduled visit will be performed at early termination.

8. STUDY INTERVENTION

8.1 Investigational Products (treatment / medical device)

Not applicable. All antibiotics are available on the Swiss market and approved by *Swissmedic.*

8.1.1 Intervention treatment

<u>Broad spectrum antibiotic prophylaxis</u>:

Single-shot combination of vancomycin 1 g intravenously with gentamicin 5 mg/kg intravenously. The continuation of prophylaxis up to three doses or the antibiotic therapy according to the medical indication will occur individually. Local antiseptic or antibiotic agents allowed.

8.1.2 Control Comparator

<u>Usual antibiotic prophylaxis:</u>

Standard antibiotic prophylaxis or current antibiotic therapy. The standard prophylaxis consists of one to three intravenous doses of cefuroxime 1.5 g intravenously; or 3 g if obesity; or vancomycin 1 g or rather clindamycin 600 mg; if allergy. Local antiseptic or antibiotic agents allowed.

8.1.3 Packaging, Labelling and Supply (re-supply)

According to *Swissmedic* guidelines. The Pharmacy of UKB supplies with all antibiotics. No study specific packing or labelling.

8.1.4 Storage Conditions

Medication is stored according to *Swissmedic* guidelines by the pharmacy of UKB.

8.2 Dose modifications

The prophylactic doses are fix in this study. For an eventual current antibiotic therapy, the doses of the antibiotic agents are selected according to the individual case and are individually determined by the treating clinicians.

8.4 Compliance with study intervention

No study specific surveillance of compliance (like diaries) with the prescribed antibiotic

prophylaxis will be implemented. Patients with recurrences / clinical persistence of local infection, patients withdrawing from the study; or patients removed from the study, will be treated according to therapeutic and surgical standards at Balgrist Hospital.

8.5 Data Collection and Follow-up for withdrawn participants

Withdrawn participants are instructed to continue therapy according to the corresponding control therapy protocol and given on whom to contact if there are any questions or concerns that arise after completing the study.

8.6 Trial specific preventive measures

None.

8.7 Concomitant Interventions (treatments)

Standard wound control for all patients will include eventual wound debridement, regular wound care with dressing changes and eventual VAC / PICO use[12] in selected cases upon surgical indication.

8.8 Study Drug Accountability

All drugs are commercial products and on stock at the UKB pharmacy. Drugs are ordered, stored and handled according to UKB pharmacy standards. No study specific drug accountability will be introduced.

8.9 Return or Destruction of Study Drug

Not applicable, commercial products.

9. STUDY ASSESSMENTS

9.1 Table of study procedures and assessment

**Study Periods**	**Screening/ Baseline**	**EOT**	**TOC**	**Follow-up only for implant surgery**
**Visit**	1	2	3	4
**Time**	-30 to 0 day	14 days (+/- 3d)	6 weeks (+/- 12d)	1 year (+/- 2 months)
In-/ Exclusion criteria	X			
Informed consent	X			
Demographics and medical history[1]	X			
Randomization	X			
(Concomitant) treatment and interventions[2]		X	X	

Adverse Events/Outcomes		X	X	X
Study End[3]			X	

[1] Age, biological sex, weight, body mass index, antibiotic prophylaxis (therapy) and duration, intraoperative microbiology (if any), indication of surgery, date of surgery, osteosynthesis material, local antibiotic or antiseptic use, diabetes and other immune suppression states, duration of VAC / PICO use, body and skin colonization with multi-resistant Gram-negative bacteria (if any).

[2] Intraoperative bacterial microbiology (if any), wound discharge, seroma, hematoma, surgical site infection, all postoperative infections, number of surgical lavages and debridement, adverse events of prophylactic antibiotic therapy and its duration.

[3] Remission, clinical and microbiological recurrences, re-operation, wound problems, adverse events of prophylactic antibiotic therapy and duration, body and skin colonization with multi-resistant Gram-negative bacteria (if any), other non-SSI infections.

9.2 Assessment of outcomes

9.2.1 Assessment of primary outcomes

During the EOT and the TOC visits:

The clinicians determine the wound healing, the adverse events and the presence or absence of clinical signs of SSI, clinically and by history. The microbiological results come from the Bacterial Laboratory which are assessed by the study team and interpreted by one of the Investigators-sponsors.

9.2.3 Assessment of other outcomes of interest

The clinicians determine and note the secondary outcomes by clinical examination and history. The investigators analyse and note the bacteriological laboratory results.

9.2.4 Assessment of safety outcomes

9.2.4.1 Adverse events

See chapter 10

9.2.4.2 Laboratory parameters

n/a. No study specific laboratory parameters will be assessed

9.2.4.3 Vital signs

n/a. No study specific vital sign measurements will be performed.

9.2.5 Assessments in participants who prematurely stop the study

Patients who withdraw consent or who, in the opinion of the investigator, are no longer able or eligible to participate in the study will be early terminated. Where possible, such patients will complete an early-termination visit to undergo all assessments applicable to the corresponding (or next) scheduled study visit. For these patients, we will record eventual adverse events, physical examination, laboratory parameters, vital signs during a follow-up period determined by clinical control, which is equally and usually up to

one-year post intervention.

9.3 Procedures at each visit for both prospective-randomized studies

At enrollment (Day 1), the investigator will prescribe the preoperative antibiotic prophylaxis based on instructions provided in the protocol according to the patient's randomization. Patients will be randomized in the ratio 1:1 into the investigational group (broad-spectrum prophylaxis) and the control group (usual prophylaxis). Each patient can be included several times with one episode for each surgical intervention in the operating theatre. The initial consent form is valid during 1 month and will be renewed in case of another inclusion following a delay of 1 month after the initial signature of the patient.

The study period only includes the following daily study visits:

- Visit 1 - Enrollment (Day 1)
- EOT (end of microbiological cultures) - Day 14 (+/- 3 days)
- TOC (clinical surgical control) - Day 42 (+/- 12 days)
- Follow-up for implant-related surgery - 1 year (+/- 2 months)

9.3.1 Screening/Pre-procedure assessment / Visit 1

If the patient agrees, he/she will be randomized by the central study nurse / research assistant. The anaesthetist (or the hospitalization unit) will administer the prophylactic regimen. The corresponding study nurse / research assistant will collect the data. In case of (presumed) infection, the surgeon will sample at least three intraoperative tissues (after the prophylaxis) for microbiological cultures.

9.3.2 Visit 2 (End of Treatment)

The EOT visit corresponds to the termination of the microbiological cultures at the IMM in Zurich. The corresponding study nurse / research assistant will collect the study-specific data from the Bacterial Laboratory and from the medical and nursing files. If the patient is discharged, he/she does not have to come physically for a clinical control. The investigators or the study nurse/research assistant might phone the patient in case of unclear information.

9.3.3 Visit 3 (Test of Cure)

The TOC visit corresponds to the usual and traditional surgical control at approximatively 4-6 weeks post-intervention. Besides the clinical controls with eventual radiological, microbiological and blood exams for clinical reasons (including for infection control purposes), the clinicians and investigators might specifically ask for eventual antibiotic-related adverse events.

9.3.4 Follow-up for implant surgery patients with implants kept in place

Usually, patient with implant-related surgery are seen for a control after one year. If this is not the case for various reasons, the study nurse/research assistant and the investigators might phone the patient for an oral follow-up information regarding the study outcomes.

9.3.4 Early Termination of Study Patients

Patients who withdraw consent or who, in the opinion of the investigator, are no longer able or eligible to participate in the study (including patients who require antibiotic therapy beyond EOT) will be early terminated. Where possible, such patients will complete an early-termination visit to undergo all assessments applicable to the corresponding (or next) scheduled study visit. Patients with recurrences / clinical persistence of local infection, patients withdrawing from the study; or patients removed from the study, will be treated according to therapeutic standards at the UKB.

10. SAFETY

10.1 Drug studies

During the entire duration of the study, all serious adverse events (SAEs) are collected, fully investigated and documented in source documents and electronic case report forms (eCRF). Study duration encompassed the time from when the participant signs the informed consent until the last protocol-specific procedure has been completed, including a safety follow-up period.

10.1.1 Treatment by specialists at UKB

All surgeries will be performed in the supervision and participation of an advanced and experienced surgeon. The antibiotic prophylaxis is ordered and / or supervised by anaesthesiologists and surgeons. The current prophylaxis of the operated study patients will be controlled by the Head of Pharmacy UKB.

10.1.2 Definition and assessment of (serious) adverse events and other safety related events

An **Adverse Event (AE)** is any untoward medical occurrence in a patient or a clinical investigation participant administered a pharmaceutical product, and which does not necessarily have a causal relationship with the study procedure. An AE can therefore be any unfavourable and unintended sign (including an abnormal laboratory finding), symptom, or disease temporally associated with the use of a medicinal (investigational) product, whether or not related to the medicinal (investigational) product.

A **Serious Adverse Event (SAE)** is classified as any untoward medical occurrence that:

- results in death
- is life-threatening
- requires in-patient hospitalization or prolongation of existing hospitalisation
- results in persistent or significant disability/incapacity
- is a congenital anomaly/birth defect

In addition, important medical events that may not be immediately life-threatening or result in death, or require hospitalisation, but may jeopardise the patient or may require intervention to prevent one of the other outcomes listed above should also usually be considered as serious.

Examples of such events are intensive treatment in an emergency room or at home

for allergic bronchospasm, blood dyscrasias or convulsions that do not result in hospitalization, or development of drug dependency or abuse.

SAEs should be followed until resolution or stabilisation. Participants with ongoing SAEs at study termination (including safety visit) will be further followed up until recovery or until stabilisation of the disease after termination.

Assessment of Causality

The investigators will make a causality assessment of the event to the study drug, based on the criteria listed in the ICH E2A guidelines:

**Relationship**	**Description**
Definitely	Temporal relationship<br>Improvement after dechallenge*<br>Recurrence after rechallenge<br>(or other proof of drug cause)
Probably	Temporal relationship<br>Improvement after dechallenge<br>No other cause evident
Possibly	Temporal relationship<br>Other cause possible
Unlikely	Any assessable reaction that does not fulfil the above conditions
Not related	Causal relationship can be ruled out
*Improvement after dechallenge only taken into consideration, if applicable to reaction	

Unexpected Adverse Drug Reaction

An “unexpected” adverse drug reaction is an adverse reaction, the nature or severity of which is not consistent with the applicable product information (e.g. Investigator’s Brochure for drugs that are not yet approved and Product Information for approved drugs, respectively).

Suspected Unexpected Serious Adverse Reactions (SUSARs)

The Sponsor-Investigator evaluates any SAE that has been reported regarding seriousness, causality and expectedness. If the event is related to the investigational product and is both serious and unexpected, it is classified as a SUSAR.

Assessment of Severity

This study uses a severity grading scale as described in the “Common Terminology Criteria for Adverse Events CTCAE Version 4.

10.1.3 Reporting of serious adverse events (SAE) and other safety related events

Reporting of SAEs

All SAEs must be reported immediately and within a maximum of 24 hours to the Sponsor-Investigator of the study. The Sponsor-Investigator will re-evaluate the SAE and return the form to the site.

SAEs resulting in death are reported to the local Ethics Committee (via local Investigator) within 7 days.

Reporting of SUSARs

A SUSAR needs to be reported to the local Ethics Committee (local event via local Investigator) within 7 days, if the event is fatal, or within 15 days (all other events).

Reporting of Safety Signals

All suspected new risks and relevant new aspects of known adverse reactions that require safety-related measures, i.e. so-called safety signals, will be reported to the Sponsor-Investigator within 24 hours. The Sponsor-Investigator will report the safety signals within 7 days to the local Ethics Committee (local event via local Investigator).

Reporting and Handling of Pregnancies

This study, all antibiotics and therapeutic surgeries, have no specific relation to pregnant or breast-feeding women and their children. The study population is likely not to reveal pregnant women. Formally, pregnant and breast-feeding women thus are not excluded from this cohort and its side studies.

Any pregnancy during the treatment phase of the study and within 30 days after discontinuation of study medication will be reported to the Sponsor-Investigator within 24 hours. The course and outcome of the pregnancy will be followed up carefully, and any abnormal outcome regarding the mother or the child should be documented and reported.

Periodic reporting of safety

An annual safety report on the participant is submitted once a year to the local Ethics Committee via the Lead Investigator. We, moreover, will perform statistical interim (futility) analysis on an annual basis.

10.1.4 Follow up of (Serious) Adverse Events

Participants terminating the study (either regularly or prematurely) with reported ongoing SAE, or any ongoing AEs of laboratory values or of vital signs being beyond the alert limit will return for a follow-up investigation. This visit will take place up to 30 days after terminating the treatment period. Follow-up information on the outcome will be recorded on the respective AE page in the CRF/eCRF. All other information has to be documented in the source documents. Source data have to be available upon request.

In case of participants lost to follow-up, efforts will be made and documented to contact the participant to encourage him/her to continue study participation as scheduled. In case of minor AE, a telephone call to the participants may be acceptable.

All new SAE or pregnancies that the investigators will be notified of within 30 days after discontinuation of study medication will be reported in appropriate report forms and in

the CRF/eCRF, if required.

Follow-up investigations may also be necessary according to the investigator's medical judgment even if the participant has no AE at the end of the study. However, information related to these investigations does not have to be documented in the CRF/eCRF, but must be noted in the source documents.

11. STATISTICAL METHODS

11.1 Main hypotheses

For the selected study population with a high risk of (antibiotic-resistant) SSI and related postoperative wound problems, alongside with the changing microbiology of the deep surgical site (open wound) a broad-spectrum prophylaxis may significantly reduce the (antibiotic-resistant) SSIs and/or the deep colonization of the operation site by these pathogens.

11.2 Determination of Sample Size

Postoperative wound problems occur in up to 5% percent of all orthopedic interventions[8]. The number of annual oncologic interventions is 120-150 surgeries. The performance of the antibiotic prophylaxis to reduce SSI in orthopedic surgery is 5-10%[1]. Per analogy, the performance of a broad-spectrum prophylaxis to reduce antibiotic-resistant intraoperative samples is at maximum 5-10%. The proportion of selection of antibiotic-resistant new bacteria in iterative surgery, surgery under antibiotic use, or pen wounds is 10%[2].

In this RCT, we perform a superiority RCT with a 10% margin and a power of 90% in favor of the broad-spectrum prophylaxis. With event-free surgeries to 95% in the broad-spectrum versus 85% in the standard arm, we need 2 x 207 episodes among the selected patient groups. At the Balgrist, we perform at least 5000 operations per year. With a conservative estimation, we see 300 surgical episodes per year (6%) that can be included in our RCT, and its stratifications. The study will last two years.

11.3 Planned Analyses

All analyses will be performed for the entire study population. In a second step, all analyses will be separately performed according to substrata of patients and surgeries: implant versus non-implant; tumour surgery, surgery under therapeutic antibiotics.

11.3.1 Interim analysis and early termination

We will perform one interim analysis after one year (+/- 2 months). If any group comparison regarding remission, wound problems, SSI and AE between the corresponding broad and narrow-spectrum prophylaxis are striking, or statistically significant, in terms of any study objective, the independent Data Monitoring committee will decide the interruption and early termination of the study (or, alternatively, to continue the study only in substrata). Otherwise, the study will finish as scheduled. The Data Monitoring committee has also the right to call on an additional interim analysis.

11.3.2 Final analyses

We will use descriptive statistics and will compare groups by the Pearson-χ^2-test or

the Wilcoxon-Ranksum-test, as appropriate. For the primary outcome parameter, univariable and multivariable results will be computed using a Cox regression analysis. Variables with a *p* value ≤0.2 in univariate analysis will be included in a stepwise forward selection process for multivariate analysis. Key variables will be checked for colinearity and interaction. The number of variables in the final model is limited to the ratio of 1 variable to 5 to 8 outcome events[20].

The intent-to-treat (ITT) population will consist of all randomized patients. Patients will be analysed according to prophylaxis group assignment. Patient disposition and baseline characteristics will be based on the ITT population.

The per-protocol (PP) population will consist of all randomized patients who complete the study and who have not deviated significantly from the protocol. All efficacy analyses will be repeated using the PP population.

11.4 Handling of missing data and drop-outs

Missing data regarding the antibiotic prescription will lead to patient dropout of the study. Drop-outs will be reported in the patients & methods section of the publication, drop-out data will be archived for a minimum of 10 years after study termination or in case of premature termination of the clinical trial. Other clinical data are so essential for the clinical therapy by itself, that we do not expect major missing data biases.

12. QUALITY ASSURANCE AND CONTROL

The Sponsor-Investigators will implement and maintain quality assurance and quality control systems with written SOPs and Working Instructions to ensure that trials are conducted, and data are generated, documented (record), and reported in compliance with the protocol, GCP, and applicable regulatory requirement(s). Monitoring and Audits will be conducted during the course of the study for quality assurance purposes.

12.1 Data handling and record keeping / archiving

Data is exclusively stored using the secured REDCap® electronic data capture tool. The PI is responsible for collection of data and possesses the screening log, where confidentiality is ensured by using participants' ID. Study IDs are distributed by REDCap® automatically in ascending order. Access authorization via Log-in (User) and password will be given by the PI to people on the staff list involved in the study as necessary. For this reason, data cannot be changed by non-authorized people. REDCap® documents every relevant processing step to ensure traceability with registration software and is secured daily via backups. All transaction logs between performing of two backups will be secured for one week while every study data in REDCap® will be secured for an unconfined time, at least for 10 years. Its data base server is allocated in highly modern rooms in Rümlang and Altstetten with protection of access. Collected data of this study is visible for inspection of independent ethic committee and authorities.

When the study is terminated, data will be stored in the same system. Data can only be accessed by defined persons that have contributed to the project. Source Data are going to be stored in the institutions PACS and KISIM system according to the institutional standard at the UKB.

12.1.1 Case Report Forms

Electronic case report forms (eCRF) will be used, one for each enrolled study participant, to be filled in with all relevant data pertaining to the participant during the study. The participation of each study participant will be documented on the Enrolment Log. For data and query management, monitoring, reporting and coding an internet-based secure data base REDCap® developed in agreement to the Good Clinical Practice (GCP) guidelines will be used. It is the responsibility of the PI to assure that all data in the course of the study will be entered completely and correctly in the respective data base. Corrections in the eCRF may only be done by the investigator or by other authorized persons. In case of corrections the original data entries will be archived in the system and can be made visible. For all data entries and corrections date, time of day and person who is performing the entries will be generated automatically. Documented medical histories and narrative statements relative to the participant's progress during the study will be maintained. These records will also include the following: originals or copies of laboratory results, which must be kept on file with the individual participant's eCRF. The investigators assure to perform a complete and accurate documentation of the participant data in the eCRF.

12.1.2 Specification of source documents

Source data will be available at the site to document the existence of the study participants and substantiate the integrity of study data collected. Source data will include the original documents relating to the study, as well as the medical treatment and medical history of the participant.

The following information (at least but not limited to) will be included in the source documents:

- Demographic data (age, sex)
- Inclusion and Exclusion Criteria details
- Participation in study and signed and dated Informed Consent Forms
- Visit dates
- Medical history and physical examination details
- Key efficacy and safety data (as specified in the protocol)
- SAEs, AEs and concomitant medication
- Results of relevant examinations
- Laboratory printouts
- Reason for premature discontinuation
- Randomization number

Source data will always be kept with the regular patient file.

The following documents are also considered source data, including but not limited to:

- SAE worksheets
- Nurse records, records of clinical coordinators
- Medical records from other department(s), or other hospital(s), or discharge letters and correspondence with other departments/hospitals, if participant visited any during the study period and the post study period.

12.1.3 Record keeping / archiving

All study data must be archived for a minimum of 10 years after study termination or premature termination of the clinical trial. Data are stored using the proprietary hospital

information system and REDCap electronic data capture tool hosted at the UKB.

12.2 Data Management System, access and back-up

Subject-related data will be stored in the research electronic data capture software REDCap. Back up will be kept on a hard drive belonging to the PI and later on stored in the archives of the UKB, as mentioned above. The PI and the co-investigators are responsible for data recording. The PI will grant the relevant personnel user rights to view and/or edit data entries by password as applicable. All edits will be automatically documented in the change history log.

12.2.1 Analysis and archiving

For data analysis, subject-related data from REDCap will be exported and analyzed in statistics software (IBM – SPSS and/or STATA, Version 14, College Station, USA). Before data export, all patient identifiers will be removed. All eCRF data will be stored for a minimum of 10 years.

12.3 Monitoring

Regular monitoring visits at the investigator's site prior to the start and during the course of the study will help to follow up the progress of the clinical study, to assure utmost accuracy of the data and to detect possible errors at an early time point. The Sponsor-Investigator organizes professional independent monitoring by the UCAR (Unit for Clinical and Applied Research) for the study.

All original data including all patient files, progress notes and copies of laboratory results must be available for monitoring. The monitor will review all or a part of the eCRFs and written informed consents. The accuracy of the data will be verified by reviewing the above referenced documents.

One monitoring visit at the investigator's site prior to the start, and once during the course of the study, will be organised by the Sponsor-Investigator. Furthermore, there will be a close-out visit at the study end. During the monitoring, all documents including source data/documents will be accessible for the monitor and all questions will be answered.

**Study period**	**Time**	**Monitoring**
Before study	Spring 2022	Monitoring will be informed about study conduct concerning data sampling and safety reporting.<br>Monitor controls if<br>• Documents are approved<br>• Documents are at site<br>• Investigators are familiar with study protocol and safety reporting<br>• Investigators know their duties and responsibilities
Interim analysis	Spring 2023	All subjects: SDV for existence and informed consent<br>First trial participant and at least 10% of trial participants recruited at the time of the visit, as far as available:

		eligibility, primary endpoint, SAEs
Study end	December 2023	Control for completeness of source data

12.4 Audits and Inspections

A quality assurance audit/inspection of this study may be conducted by the competent authority or CEC, respectively. The quality assurance auditor/inspector will have access to all medical records, the investigator's study related files and correspondence, and the informed consent documentation that is relevant to this clinical study. The investigator will allow the persons being responsible for the audit or the inspection to have access to the source data/ documents and to answer any questions arising. All involved parties will keep the patient data strictly confidential.

12.5 Confidentiality, Data Protection

Direct access to source data may be granted in the case of monitoring, audit or inspections. All personnel must treat patient data as confidential. As far as possible, encoded data will be used. Only persons listed on the staff list have access to the source data.

12.6 Storage of biological material and related health data

All health-related patient data will be stored and archived in the data capture software REDCap. Patient-source data will be registered using subject identifiers. After full data analysis, all subject identifiers will be erased. Patient-source data may still be saved in the patient’s medical record. Collection, disclosure, storage of patient-related data is carried out in accordance with Swiss data protection regulations and the Human Research Act. A requirement is the informed consent of every subject prior to inclusion in the clinical trial.

13. PUBLICATION AND DISSEMINATION POLICY

After the statistical analysis of this trial the sponsor will make every endeavour to publish the data in (a) medical journal(s), to be able to communicate the results to healthcare professionals, the public and other relevant groups. All participants will be sent a free copy of the published article. There will not be any publication restriction and we plan to sort at least three major publications. We will also present preliminary results in national, regional, and international scientific meetings.

All investigators indicated in this protocol, and eventually additional colleagues participating in the future, will be co-authors of this study according to their individual contributions.

14. FUNDING AND SUPPORT

This project is funded by the Research Funds of University Hospital Balgrist. The initial internal financing of UKB will be 8’000 Swiss Francs. In addition, the investigators will demand for grants to obtain more study funding.

15. INSURANCE

The standard Balgrist research insurance is applicable. Insurance police Nr. 14.050.565 Winterthur Versicherung. Any damage developed in relation to study participation is covered by this insurance. So as not to forfeit their insurance cover, the participants themselves must strictly follow the instructions of the study personnel. Participants must not be involved in any other medical treatment without permission of the principal investigator (emergency excluded). Medical emergency treatment must be reported immediately to the investigator. The investigator must also be informed instantly, in the event of health problems or other damages during or after the course of study treatment. The investigator will allow delegates of the insurance company to have access to the source data/documents as necessary to clarify a case of damage related to study participation. All involved parties will keep the patient data strictly confidential. A copy of the insurance certificate will be placed in the Investigator's Site File and the trial master file.

16. REFERENCES

1) Uçkay I, Hoffmeyer P, Lew D, Pittet D. Prevention of surgical site infections in orthopaedic surgery and bone trauma: state-of-the-art update. J Hosp Infect. 2013 May;84(1):5-12.
2) Wuarin L, Abbas M, Harbarth S, et al. Changing perioperative prophylaxis during antibiotic therapy and iterative debridement for orthopedic infections? PLoS One. 2019 Dec 18;14(12):e0226674.
3) Davat M, Wuarin L, Stafylakis D, et al. Should antibiotic prophylaxis before orthopedic implant surgery depend on the duration of pre-surgical hospital stay? Antimicrob Resist Infect Control 2018 Nov;7:131.
4) Jamei O, Gjoni S, Zenelaj B, et al. Which Orthopaedic Patients Are Infected with Gram-negative Non-fermenting Rods? J Bone Jt Infect 2017 Jan 15;2(2):73-76.
5) Uçkay I, Harbarth S, Ferry T, et al. Meticillin resistance in orthopaedic coagulase-negative staphylococcal infections. J Hosp Infect 2011 Nov;79(3):248-53.
6) Müller D, Kaiser D, Sairanen K, et al. Antimicrobial Prophylaxis for the Prevention of Surgical Site Infections in Orthopaedic Oncology - A Narrative Review of Current Concepts. J Bone Jt Infect 2019 Oct 15;4(6):254-263.
7) Rod-Fleury T, Uçkay I. Microbiological Particularities of Surgical Site Infections in Oncologic Orthopedic Surgery Compared to Non - Oncologic Surgery-Single Center Experience and Literature Review. Clin Surg. 2019; 4: 2443.
8) Dunkel N, Pittet D, Tovmirzaeva L, et al. Short duration of antibiotic prophylaxis in open fractures does not enhance risk of subsequent infection. Bone Joint J-2013 Jun;95-B(6):831-7.
9) Uçkay I, Agostinho A, Belaieff W, et al. Noninfectious wound complications in clean surgery: epidemiology, risk factors, and association with antibiotic use. World J Surg. 2011; 35(5):973-80.
10) Ertuğrul B, Uçkay I, Schöni M, et al. Management of diabetic foot infections in the light of recent literature and new international guidelines. Expert Rev Anti Infect Ther 2020 Apr;18(4):293-305.

11) Al-Mayahi M, Cian A, Lipsky BA, et al. Administration of antibiotic agents before intraoperative sampling in orthopedic infections alters culture results. J Infect. 2015 Nov;71(5):518-25.

12) Karaca S, Cikirikcioglu M, Uçkay I, et al. Comparison of vacuum-assisted closure device and conservative treatment for fasciotomy wound healing in ischaemia-reperfusion syndrome: preliminary results. Int Wound J 2011 Jun;8(3):229-36.

13) Mangram AJ, Horan TC, Pearson ML, Silver LC, Jarvis WR. Guideline for prevention of surgical site infection, 1999. Infect Control Hosp Epidemiol 1999,20:250-78.

14) Uçkay I, Kressmann B, Di Tommaso S, et al. A randomized controlled trial of the safety and efficacy of a topical gentamicin-collagen sponge in diabetic patients with a mild foot ulcer infection. SAGE Open Med 2018 May 13;6:2050312118773950.

15) Uçkay I, Kressmann B, Malacarne S, et al. A randomized, controlled study to investigate the efficacy and safety of a topical gentamicin-collagen sponge in combination with systemic antibiotic therapy in diabetic patients with a moderate or severe foot ulcer infection. BMC Infect Dis 2018 Aug 2;18(1):361.

16) Cui Q, Mihalko WM, Shields JS, et al. Antibiotic-impregnated cement spacers for the treatment of infection associated with total hip or knee arthroplasty. J Bone Joint Surg Am 2007 Apr;89(4):871-82.

17) Uçkay I, Berli M, Sendi P, et al. Principles and practice of antibiotic stewardship in the management of diabetic foot infections. Curr Opin Infect Dis 2019 Apr;32(2):95-101

18) Harbarth S, Samore MH, Lichtenberg D, et al. Prolonged antibiotic prophylaxis after cardiovascular surgery and its effect on surgical site infections and antimicrobial resistance. Circulation 2000 Jun 27;101(25):2916-21.

19) Vauthey L, Uçkay I, Abrassart S, et al. Vancomycin-induced DRESS syndrome in a female patient. Pharmacology. 2008;82(2):138-41.

20) Vittinghoff E, McCulloch CE. Relaxing the rule of ten events per variable in logistic and Cox regression. Am J Epidemiol 2007 Mar 15;165(6):710-8.
