# Supplementary material for: Broad-spectrum antibiotic prophylaxis in tumor and infected orthopedic surgery—the prospective-randomized, microbiologist-blinded, stratified, superiority trials: BAPTIST Trials
Source: Trials. 2024 Jan 19;25:69. doi: 10.1186/s13063-023-07605-5 (PMC10799415; doi:10.1186/s13063-023-07605-5)
Supplement: Supplementary file 3 — Additional file 3: Supplementary file 3. Model consent form. [file 13063_2023_7605_MOESM3_ESM.docx]

**Patient information**

**Broad-spectrum antibiotic prophylaxis versus standard antibiotic prophylaxis during orthopedic operations with an increased risk of postoperative infections with resistant germs**

This study is organized by: Prof. Dr. med. Ilker Uçkay, Balgrist University Hospital

Dear Patient,

You will soon undergo orthopedic surgery. Routinely, an antibiotic prophylaxis takes place in practically all orthopedic operations. The anesthetists, or the nurses on the hospitalization ward, will administer one or more prophylactic antibiotics before, during, or after surgery; to reduce the risk of possible subsequent infections. This standardized prophylaxis is based on scientific evidence, established, and protocolled for the majority of operations worldwide.

Usually, the prophylaxis consist of a narrow-spectrum antibiotic agents. However, for patients with particular risks for postoperative infections and infections due to antibiotic-resistant bacteria, the question of a possibly better prophylaxis still remains open. We would therefore like to ask you if you would like to participate in this clinical study, which is presented below.

**1. Aim of the study**

We want to find out if a very broad antibiotic prophylaxis with vancomycin and gentamicin (with single doses each) would be superior to the standard prophylaxis (cefuroxime, clindamycin) in special situations. These "risk situations" are colonization with several antibiotic-resistant bacteria, tumor surgery, open fractures, spinal surgery in patients with multiple comorbidities (American Society of Anesthesiologists classification ≥ 3 points), skin colonization with multidrug-resistant germs, or for patients who are already under antibiotic therapy.

**2. Selection**

All patients who meet the above-mentioned definitions of extraordinary situations and can come to the usual, one-time, surgical follow-up in 4 - 6 weeks can participate. Also, you must be at least 18 years old. Pregnant women, women who are breastfeeding, and people with documented allergies to vancomycin or gentamicin are not allowed to participate.

**3. General information**

Perioperative antibiotic prophylaxis is scientifically established for the vast majority of surgical operations; to reduce the risk of postoperative infections. Around the world, one, two or three doses of intravenous antibiotics are administered (cefuroxime, clindamycin or vancomycin for penicillin allergy). These common antibiotics target the common skin germs. However, there are situations when previous antibiotic intake, open contaminated wounds, many concomitant diseases, or tumors could change the skin flora and the skin could be colonized with several resistant germs. In these selected situations, it remains unclear whether a short, but broader antibiotic prophylaxis, leads to a better risk reduction of many postoperative infections (in the surgical area or elsewhere in the body, for example in the urine). However, such an expensive broad-spectrum antibiotics should only be used if there is absolutely necessary. These broad-spectrum antibiotics serve as a reserve for the treatment of infections with resistant pathogens. In prophylactic use, and contrary to the use during treatment, the risk of the development of antibiotic resistant among the bacteria in the body, should be negligible.

**4. Procedure**

If you agree to participate in this study, you will either receive the protocolized standard prophylaxis (possibly adapted to eventual penicillin allergy), or you will receive the extended, broader antibiotic prophylaxis with a combination of vancomycin (1 g) and gentamicin (5 mg per kilogram of body weight). Additional antibiotic administrations, such as ongoing therapies, local antibiotics, etc., remain at the discretion of the treating surgeon.

Regardless of this study, you will be called up for surgical control in about 4 - 6 weeks; perhaps even earlier, depending on the clinical course. In the case of implant-associated surgeries, there is usually an annual surgical check-up. The 6-week follow-up (and the annual follow-up) are the only controls where you will be interviewed for the study. Otherwise, the study team will have access to your medical data during the study period. The study itself does not incur any additional follow-up appointments, examinations or costs.

You may be excluded from the study in your best interest. This can happen if, for example, you have a documented allergy to vancomycin or gentamicin; or if, from the point of view of your treating physician, you do no longer qualify to participate.

**5. Benefits**

You may be able to benefit from a broader antibiotic prophylaxis, but we cannot guarantee it. In this study, half of the participants will receive the standard, and the other half the broad-spectrum prophylaxis. However, there is a benefit for future patients, or at least a gain in knowledge: These results can be important in order to optimize prophylaxis for future patients.

**6. Rights**

You participate voluntarily. If you chose not to participate, you have no disadvantages regarding your future care. The same applies if you revoke your consent at a later date. You have this option at any time. You do not have to justify any revocation of your consent or withdrawal from the study. In the event of a revocation, the data collected up to this point in time will be evaluated. Subsequently, we will anonymize your study data, i.e. we will delete your name on it. No one will be able to know that the data comes from you.

**7. Obligations**

As a participant, it is important that you

- adhere to the guidelines and prescriptions of your attending physician.
- Inform your doctor about the course of the disease and report new symptoms, new complaints or changes in well-being.
- Inform the investigator about concomitant treatment and therapies with another physicians and changes in regular medication.

**8. Risks and burdens for the participants**

A conceivable disadvantage of the broader antibiotic prophylaxis can be increased side effects, since the antibiotics vancomycin and the gentamycin are broader in their activity spectrum than the standard cefuroxime. The classic side effects with a single administration may be skin rashes, allergies or nausea.

**9. Other treatment options**

You are not obliged to participate in this study. If you do not participate, you will receive the standard prophylaxis protocol, which also corresponds to the worldwide standard.

**10. Findings from the study**

During the study, the investigator will inform you of any new findings that may affect the usefulness of the study or your safety and thus your consent to participate in the study. You will receive the information verbally and in writing. You will be informed in the event of incidental findings that may contribute to the prevention, detection and treatment of existing or expected future diseases. If you do not wish to be informed, please speak to your investigator.

**11. Confidentiality of data and samples**

We will collect your personal and medical data for this study. Very few professionals will see your encrypted data, and only to perform tasks within the scope of the study. Encryption means that all reference data that could identify you (name, date of birth) is deleted and replaced by a key. The key list always remains at Balgrist University Hospital. Therefore, those people who do not know the key will not be able to draw any conclusions about your person. In the case of a publication, the summarized data is therefore not traceable to you as an individual. We will not publish your name anywhere, in any report, publication, print or on the Internet.

It is possible that this study will be reviewed by the responsible ethics committee, the medicines authority *swissmedic* or by the institution that initiated the study. They all make sure that the rules are followed and that your safety is not compromised. The head of the study may be required to disclose your personal and medical information for such checks. All persons involved in the study must maintain absolute confidentiality.

**12. Withdrawal**

You can withdraw from the study at any time if you wish. The data collected up to this point is still evaluated in encrypted form, otherwise the entire project loses its value. After evaluation, your data will be completely anonymized, i.e. your key mapping will be destroyed so that no one can know that the data originally came from you.

**13. Compensation for participants**

You will not receive any remuneration for participating in this clinical trial. No remuneration is provided. You or your health insurance company will not incur any costs for participation.

**14. Liability**

If you suffer damage as a result of the study, the institution responsible for conducting the study is liable. The procedure is regulated by law. Balgrist University Hospital has taken out insurance with AXA Insurance, General-Guisan-Straße 40, 8400 Winterthur, Switzerland, in order to be able to pay for liability in the event of damage. In the case of damage that is attributable to an approved remedy used in accordance with the medical standard or that would have occurred if a conventional therapy had been used, the same liability regulations apply as for treatment outside of a study. If you have suffered a claim, please contact the contact persons mentioned below or the insurance company mentioned above.

**15. Financing of the study**

This study will be funded by the Balgrist University Hospital's Scientific Fund and possibly by other future sources such as research funding from foundations and authorities.

**16. Contact person**

In case of ambiguity, emergencies, unexpected or adverse events that occur during the study or after its completion, you can always contact the contact persons below:

| Prof. Dr. med. Ilker Uckay  Infectiology, Head of Clinical Research  Balgrist University Hospital  Forchstrasse 340, 8008 Zurich  Phone: 044 386 11 11  Email: ilker.uckay@balgrist.ch |
| --- |

**Consent**

**Written declaration of consent for participation in a study project**

Please read this form carefully. Please ask if there is something you do not understand or want to know. Your written consent is required for participation.

| **Title of the study:** | "BAPTIST Trials"  Broad-spectrum Antibiotic Prophylaxis in Tumor and Infected Orthopedic Surgery - the prospective-randomized, microbiologist-blinded, stratified, superiority Trials |
| --- | --- |
| **Responsible institution:** | Balgrist University Hospital |
| **Place of implementation**: | Balgrist University Hospital  Forchstrasse 340  CH-8008 Zurich |
| **Investigators in charge at the study site:** | PD Dr. Daniel Müller, Prof. Dr. Ilker Uçkay |
| **Participant:** Surname and first name:  Date of birth: | female  male |

- I was informed verbally and in writing by the undersigned investigator about the purpose, the course of the project, about possible advantages and disadvantages as well as about possible risks.
- I participate in this project voluntarily and accept the content of the written information submitted on the above-mentioned project dated 19.04.2022, version 1.0.
- I had plenty of time to make my decision.
- My questions related to participation in this project have been answered.
- I will keep the written information and receive a copy of my written declaration of consent.
- I am informed that insurance will cover the damage to health if such damage occurs in the course of the studies.
- I agree that the responsible experts of the project management / client of the study and the ethics committee responsible for this project may inspect my unencrypted data for testing and control purposes, but in strict compliance with confidentiality.
- In the event of study results or incidental findings that directly affect my health, I will be informed. If I don't want that, I'll inform my investigator.
- I understand that my health-related and personal data can only be shared in encrypted form for research purposes for this study project.
- I agree that if I continue to be treated outside the site, the investigator / project manager may contact the treating physicians to request follow-up treatment data relevant to the study.
- I am participating in this study on a voluntary basis. I can withdraw from participation at any time and without giving reasons, without having any disadvantages in further medical treatment / care. The data and samples collected up to that point will still be used for evaluation of the study.
- I am aware that the requirements and restrictions stated in the patient information must be adhered to during the study. In the interest of my health, the investigator may exclude me from the study.

| Place, date | Participant's signature |
| --- | --- |

**Confirmation from the investigator:**

I hereby confirm that I have explained the nature, significance and scope of the study to this participant. I assure you that I will comply with all obligations in connection with this study in accordance with applicable law. If, at any time during the conduct of the study, I learn of any aspects that could affect the participant's willingness to participate in the study, I will inform him/her immediately.

| Place, date | Surname and first name Investigator |
| --- | --- |
|  | Signature of the investigator |
